# Increasing aridity, temperature and soil pH induce soil C-N-P imbalance in grasslands

**DOI:** 10.1038/srep19601

**Published:** 2016-01-21

**Authors:** Feng Jiao, Xin-Rong Shi, Feng-Peng Han, Zhi-You Yuan

**Affiliations:** 1 State Key Laboratory of Soil Erosion and Dryland Farming on the Loess Plateau, Institute of Soil and Water Conservation, Northwest A&F University, Yangling, Shaanxi 712100, China; 2 Institute of Soil and Water Conservation, Chinese Academy of Science and Ministry of Water Resource, Yangling, Shaanxi 712100, China

## Abstract

Due to the different degrees of controls exerted by biological and geochemical processes, climate changes are suggested to uncouple biogeochemical C, N and P cycles, influencing biomass accumulation, decomposition and storage in terrestrial ecosystems. However, the possible extent of such disruption in grassland ecosystems remains unclear, especially in China’s steppes which have undergone rapid climate changes with increasing drought and warming predicted moving forward in these dryland ecosystems. Here, we assess how soil C-N-P stoichiometry is affected by climatic change along a 3500-km temperate climate transect in Inner Mongolia, China. Our results reveal that the soil from more arid and warmer sites are associated with lower soil organic C, total N and P. The ratios of both soil C:P and N:P decrease, but soil C:N increases with increasing aridity and temperature, indicating the predicted decreases in precipitation and warming for most of the temperate grassland region could lead to a soil C-N-P decoupling that may reduce plant growth and production in arid ecosystems. Soil pH, mainly reflecting long-term climate change in our sites, also contributes to the changing soil C-N-P stoichiometry, indicating the collective influences of climate and soil type on the shape of soil C-N-P balance.

Aridity has increased over most land areas since 1950^1^,^2^ and this widespread drying trend has been enhanced by recent global warming^3^. In this century, global drought areas could double due to the decreased precipitation and increased evaporation, exacerbating processes that lead to land degradation and desertification^2^,^4^,^5^. Meantime, global warming will continue for centuries to come, likely increasing the risk of both floods and droughts^3^. In particular, significant warming that occurs mostly in arid and semi- arid regions will make drought worse in these regions^6^. Given the importance of water availability and temperature on biological and geochemical processes, the predicted dryer and warmer climate will greatly alter biomass accumulation, decomposition and storage in different ways, and likely disrupt the biogeochemical cycles of C, N and P^7^, negatively affecting the provision of key services provided by these ecosystems^8^,^9^,^10^.

Concentrations of elements such as C, N and P in soils do not necessarily change in unison with increasing aridity and temperature, possibly leading to elemental imbalance that negatively affects plant growth and production. In spite of numerous studies on the responses of soils and plants to drought and warming, their effects on elemental balance have received very little attention. Microelements of C, N and P are vital for ecosystem processes, but little is known about their response to drought and warming. With increasing drought and temperature, the biological processes that drive C and N inputs and fluxes in ecosystems may be impaired due to moisture limitation at warmer conditions, resulting in soil C and N decline. In contrast, the availability of soil P is less affected by biological processes but more affected by mechanical rock weathering. The different direction and magnitude of C, N and P in response to drought in warm environment could result in a C-N-P imbalance. However, the effects of increasing drought and temperature on C:N:P ratios in terrestrial ecosystems remain unclear. Indeed, no studies have measured soil C:N:P ratios in response to experimental drought or warming treatments in fields. There are a few studies on plant C:N:P ratios but the reported results are inconclusive for the ratios’ response to drought and warming^11^.

Ecological stoichiometry, the study of balance of energy and multiple chemical elements in biological systems ranging from molecules to ecosystems, is a fundamental concept in ecology. It provides a powerful tool for us to advance our understanding of biological processes and nutrient cycling in terrestrial ecosystems^12^,^13^. In natural habitats, an organism’s C:N:P stoichiometry can be affected by both biotic and abiotic factors, including the predicted global changes such as increasing atmospheric CO_2_ and climate change^7^,^11^. Global change-induced variations in C:N:P stoichiometry might alter ecosystem structure and function by affecting producer-consumer relationships and/or competition among species^11^.

Drylands (arid, semi-arid and dry sub-humid ecosystems), covering about 41% of the Earth’s land surface and hosting over 38% of the global population, have always experienced drought. Despite much attention paid to the climate-induced decoupling of biogeochemical cycles and nutrient imbalance in soils and plants^8^,^14^,^15^,^16^,^17^,^18^,^19^, our understanding remains limited on how water shortage affects soil C-N-P balance. This is especially true in China’s grasslands; they cover more than 40% of the total land area of the country and these temperate grasslands in particular have experienced increased drought and warming over 30 years^20^. From east to west in northern China’s grasslands, the precipitation decreases but the temperature increases gradually and vegetation changes from meadow steppe, typical steppe to desert steppe as aridity increases. Over the long term it is climate that shapes the physical landscape and determines where various ecosystems can exist. Along the precipitation/temperature gradient, vegetation and soil vary in response to the long-term effects of climate. The processes that affect soil C, N and P availability are also expected to change accordingly, but in different ways, thus resulting in changing C:N:P ratios. The grassland’s services such as providing production of food, wood and biofuels, and to offset the emission of greenhouse gasses^21^,^22^, might be greatly negatively affected by this changing C-N-P balance. Despite the importance of grasslands, it is still largely unknown how the predicted increased drought and warmer conditions will affect soil C-N-P balance. In this study, we evaluated how increasing aridity and temperature influences soil C-N-P balance through analysis of collected soil samples from 65 grassland sites along a precipitation gradient from meadow steppes, to typical steppes, and to desert steppes in northern China. Aside from climate, we also determined how soil C-N-P balance varied with soil types and plant vegetation, both of which mostly reflect the long-term effect of climate change.

Because C and N availability is primarily linked to biological processes but available P is derived mainly from mechanical rock weathering and, to a lesser extent, from the organic matter decomposition^23^, we hypothesized that increasing aridity, especially at warm conditions could induce moisture limitation and lead to a decline of soil C and N. In contrast, soil P availability was hypothesized to be less affected by climate. Due to the long-term influence of climate on soils, we also hypothesized that soil C, N and P were associated with soil taxa and acidity/alkalinity.

## Results

Along this precipitation and temperature gradient, soil organic C, total N, total P, pH, available N and P varied in 2013 at 0.06 ~ 5.87%, 0.01 ~ 0.52%, 0.13 ~ 0.94%, 6.1 ~ 8.5, 2.5 ~ 23.4 mg N kg^−1^, and 0.9 ~ 9.1 mg P kg^−1^, respectively, in the soil profile to 30 cm depth (Table 1). Plotting soil nutrients against aridity index revealed significant relationships (Supplementary materials, Figs S1-S4). Soil organic C, total N, available N and total P all decreased with aridity from dry sub-arid, to semi-arid, to arid sites. In contrast, soil pH increased with aridity. Soil available P was not significantly affected by aridity. With increasing temperature from cool to warm sites, soil organic C, total N, available N and total P also decreased. In contrast, soil pH and available P increased with temperature. Reflecting the long-term effect of climate change, soil pH increased with increasing aridity and temperature, tended to have negative effects on soil organic C, total N, available N and total P. The variables of soil C, N and P were closely associated with each other and also associated with the latitude and longitude coordinates of the studied sites (Supplementary materials, Figs S5-S16).

**Figure 1:**
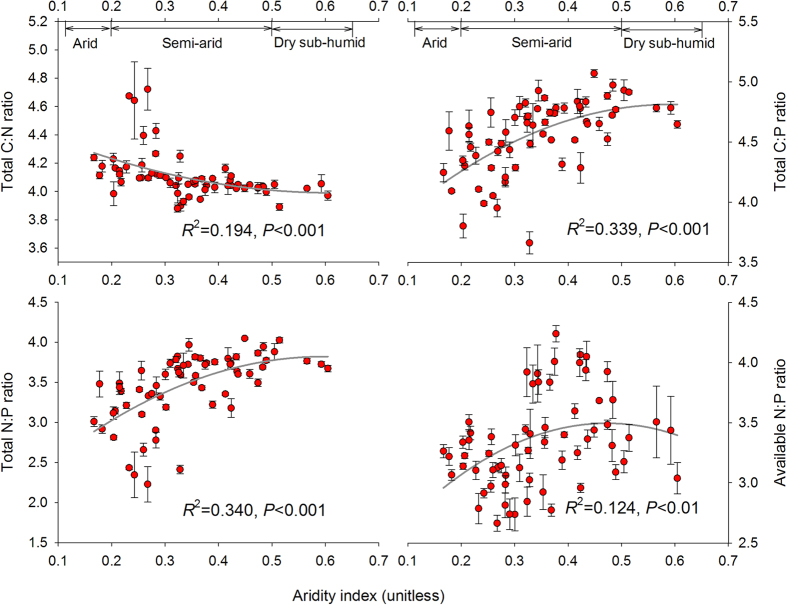
Relationships between aridity index and the ratios of soil C, N and P. Aridity index is the ratio of precipitation to potential evapotranspiration. The solid dark grey lines represent the fitted quadratic regressions. The ratio values are log-transformed. *R*^2^, proportion of variance explained. Generalized climate classification scheme for aridity values on the top is based on UNEP^51^.

**Figure 2:**
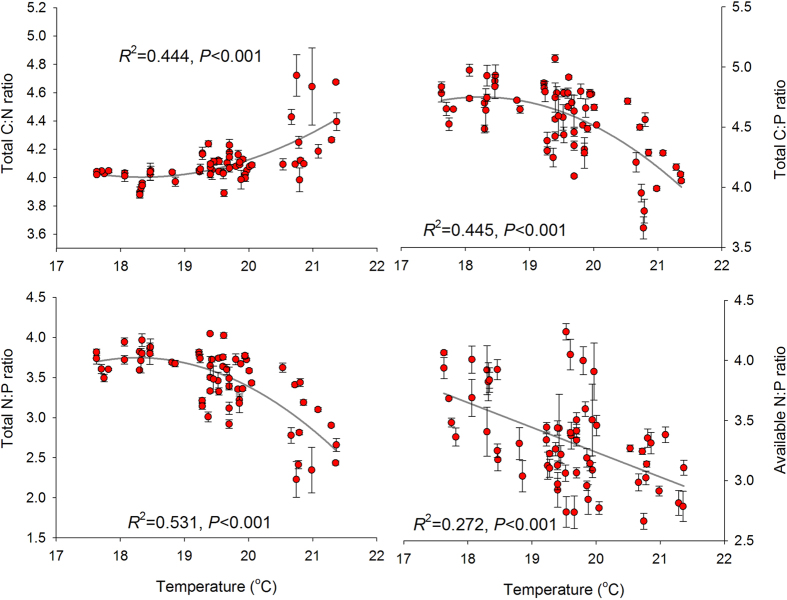
Relationships between mean temperature from May to August, 2013 and the ratios of soil C, N and P. The solid dark grey lines represent the fitted quadratic regressions. The ratio values are log-transformed. *R*^2^, proportion of variance explained.

**Figure 3:**
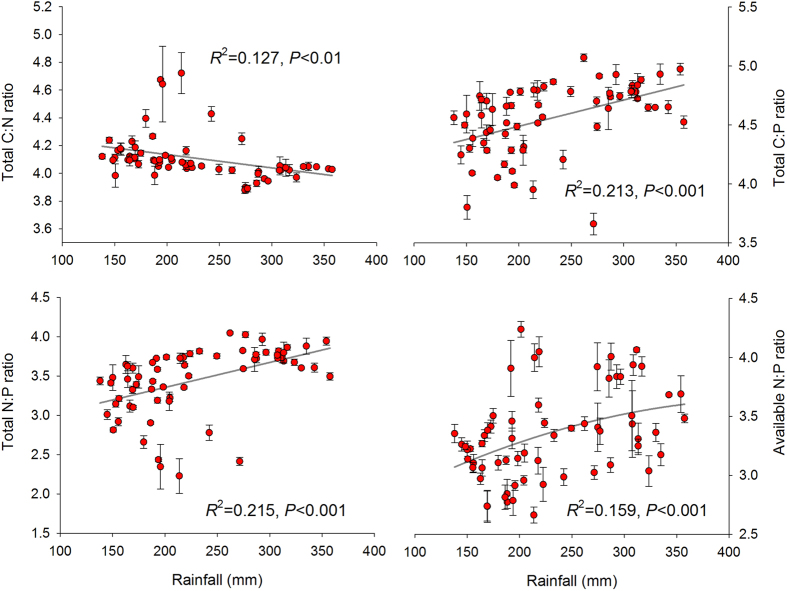
Relationships between total rainfall from May to August, 2013 and the ratios of soil C, N and P. The solid dark grey lines represent the fitted quadratic regressions. The ratio values are log-transformed. *R*^2^, proportion of variance explained.

**Figure 4:**
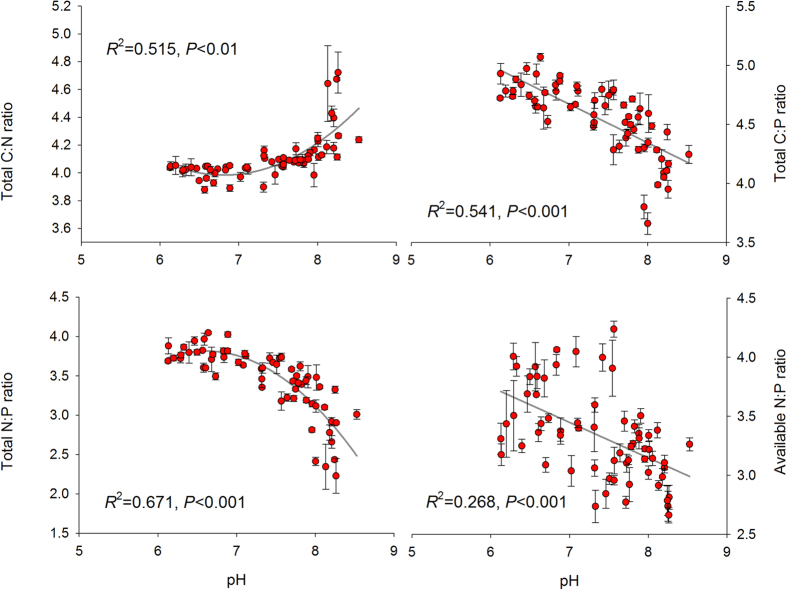
Relationships between soil pH values and the ratios of soil C, N and P. The solid dark grey lines represent the fitted quadratic regressions. The ratio values are log-transformed. *R*^2^, proportion of variance explained.

For the soil C-N-P balance, the ratios of soil C:N, C:P and N:P based on soil total N and P concentrations along this aridity and temperature gradient, varied between 7 ~ 67, 5 ~ 118 and 0.2 ~ 11 respectively. Soil available N:P ratio, calculated from available N and P, varied from 0.5 to 17.7. We found soil C:N increased, but C:P decreased quadratically with aridity. Negative quadratic relations were observed between aridity and soil total N:P ratios. A such negative quadratic relationship was also significant when available N and P were considered instead of total N and P (Fig. 1). Soil C:P, total and available N:P declined while soil C:N increased with temperature (Fig. 2). Rainfall revealed similar effect on soil C:N:P as aridity along the studied sites (Fig. 3). Soil C:N:P was closely related to soil pH values which positively affected soil C:N but negatively affected soil C:P and N:P ratios (Fig. 4).

**Figure 5:**
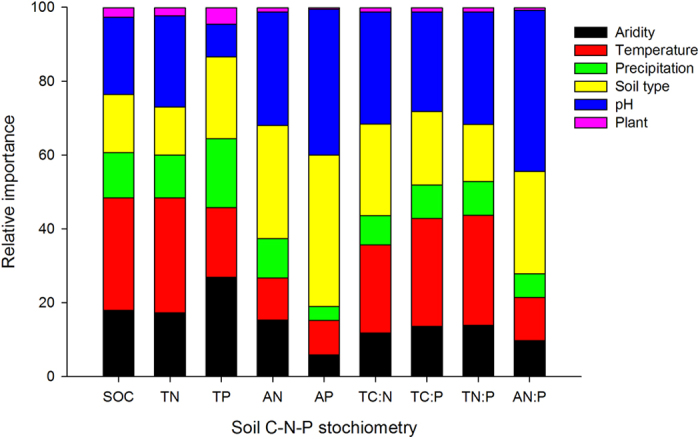
The relative influence of climatic and soil variables and plant cover on soil C, N and P. The relative importance values add to 100% for each model. In each model, predictors with large relative importance values have greater explanatory powers. See abbreviations in Table 1.

Climate data (temperature and precipitation) collectively explained 34 ~ 77% of the variation in soil organic C, total N and P and their stoichiometric ratios (Table 2). The soil type was also a strong driver over soil C:N:P ratios. Soil pH accounted for between 15 ~ 64% of the influence on soil C, N, P and their stoichiometric ratios. However, only a small fraction of soil C:N:P ratios had associated with the plant cover. When the data of soil types and pH values were also taken into account, the climate variables accounted for a partial 32 ~ 80% of the variation in soil C, N, P and their stoichiometric ratios. Climatic data (temperature and precipitation) combined with soil data (types and pH) and plant cover explained surprisingly high proportions of soil C:N:P variation: 81, 83 and 42% of the variation in soil C N, P concentrations, and 46, 67 and 67% of the variation soil C:N, C:P and N:P, respectively (Table 2). Climatic variables and soil pH generally had the major contribution to the variations in soil C:N:P ratios (Fig. 5).

## Discussion

Our findings suggest that aridity, temperature and soil acidity/alkalinity are important drivers of soil stoichiometric C, N and P in our studied sites. Because natural soil acidity/alkalinity mostly reflects long-term change of climate, our results highlighted the abiotic controls over the soil C-N-P balance in drylands. Given that drylands are more N- and P-limited than other ecosystems^24^, the decreases of soil C, N and P with aridity and temperature revealed in our analyses imply that the increasingly severe droughts and warming in drylands^2^ might lead to more severe N and P depletion in arid and warm regions, especially in the most arid and warm sites of the west end of northern China’s temperate grasslands.

In our study, we found that soil C, N and P all decreased with increasing aridity and warming. No data for soil C, N and P in response to drought or warming treatments are available so far for comparison. However, studies on plants reveal that drought treatments could enhance the concentrations of C and N but reduced the concentration of P in the bryophyte *Hypnum cupressiforme* Hedw. growing in a Mediterranean forest^25^. Because plant nutrients may not always reflect the nutrient availability in soils, how soil C, N and P response to drought and warming treatment in Mediterranean forests and whether the responses are similar to our results remains unclear. The aridity- and warming-related decline of soil N has been confirmed by natural abundance of N isotopes (δ^15^N) along a precipitation/temperature gradient in Inner Mongolian grasslands^26^. However, adding water treatments in the same ecosystem reduced soil C and N but did not affect soil P^27^. Experimental warming also revealed that increasing soil temperature could enhance soil N and P availability in grasslands^28^,^29^,^30^,^31^, indicating differing responses of soil nutrients to drought/warming between short-term manipulative experiments and long-term gradient observations.

The increase of soil C:N and decrease of soil N:P with aridity in our study were similar to the drought effects on a bryophyte in Mediterranean forest^25^. The C:N and C:P ratios of the leaves of Mediterranean shrubs and trees were also enhanced by drought^32^. Similar to our results, soil N:P ratios were found to be greater in meadow steppe than in desert steppe^33^ in Inner Mongolia. However, no effect of drought on bryophyte C:P ratios in Mediterranean forest was also found^25^. Similarly, the C:N ratios in the roots of *Quercus ilex*, a deciduous tree species, were reduced by drought treatment in semi-arid areas in the Mediterranean Basin^34^. Drought has been found to increase the C:N ratio in temperate heathlands^35^ but decrease the C:N ratio in wet-temperate ecosystems^11^, suggesting that the responses of stoichiometric ratio to drought may differ among ecosystem types and species-specific.

Similar to our soil results, experimental warming was found to enhance C:N and C:P but reduce N:P ratios in plant tissues^36^,^37^,^38^. However, stoichiometric N:P in plant tissues was also found to decline in warming experiments^39^. A global meta-analysis of manipulative experiments revealed that experimental warming tends to reduce plant C:N and enhance C:P and N:P^7^, again suggesting differences of responses between soils and plants, and differences between long-term and short-term responses.

Decreasing soil C and N with aridity can be attributed to the aridity-induced decline of soil water availability and vegetation cover, both of which directly or indirectly affect C- and N-related processes, particularly biological processes such as photosynthesis, atmospheric N fixation, and the activity of microbes and soil enzymes^40^,^41^,^42^. The increasing temperature with aridity might worsen the drought effects on soil C, N and P availability. In addition, reduced vegetation cover, together with increased temperature, enhances soil drying, promoting soil erosion that can remove fine, nutrient-rich particles such as clay^40^. These processes can result in an aridity-related soil N decline although low rainfall and high evaporation in arid sites may prevent available nutrients from being washed out of the soil profile. Given the strong photosynthesis vs. N relationship^43^,^44^,^45^, the aridity-induced reductions in N availability will limit plant production capacity that could have mitigated the rise of atmospheric CO_2_ increase in a negative feedback, thus leading to a warmer world.

Similar to the soil total N pattern, soil total P along our 3500-km transect sites also decreased with aridity and warming. However, soil available P was not affected by aridity. With increasing aridity, biological weathering, i.e., the organic matter decomposition, decreases^46^, but mechanical rock weathering may increase^42^,^47^. The non-significance of the overall effect of aridity on inorganic P in soils along our studied sites indicates that the two P-related processes offset partly each other. The decline of total P with aridity without increases in soil available P suggests increasing rock weathering, which is a physical process and provides more available P than organic matter decomposition, is the dominant driver of the non-change in soil P availability. In addition, reduced P loss through leaching due to low rainfall and high evaporation may be another driver of changes in P availability in arid sites. Our findings suggest that the levels of total P are expected to increase as soils become drier and erode more. Along our 3500-km transect from meadow steppe to desert steppe, both plant above- and belowground biomass declines with aridity and warming, suggesting that N and P sequestrated by plant biomass also decreases with aridity and warming, thus promoting high available N and P pools in soils, but in differing extent (supplementary materials, Figs S1 and S2). The different trends of soil N and P availability with aridity and warming suggest that the decline of plant production in our arid and warm sites is attributed to the changes in soil N rather than P availability and therefore N is likely the primary limiting nutrients in arid and warm systems.

The trend of aridity-related soil P is different from that by Delgado-Baquerizo, *et al.*^8^ who reported that total P was not affected, but inorganic P was positively affected by aridity across all continents except Antarctica. The responses of soil nutrients, particularly P, to aridity at a world scale, therefore, cannot be applied to regional scale. The difference in inorganic P can be attributed to the different pattern of its parent total P which significantly declines with aridity in our study, but does not change in the study by Delgado-Baquerizo, *et al.*^8^ at a larger geographic scale. Soil nutrients indeed reflect the overall effect of aridity in the balance of gains (weathering, atmospheric deposition, N fixation and organic matter decomposition) and losses (erosion, leaching and gaseous losses), whether biological or geochemical.

Our analyses reveal that soil C-N-P balance is strongly affected by temperature, apart from rainfall. Soil C, N and P decrease with decreasing precipitation but increasing temperature, suggesting that global warming will exacerbate the drought-induced C-N-P shortage and imbalance due to the co-variation of temperature and precipitation. Our results also demonstrate that soil properties, especially soil pH values, are strong drivers of soil C, N and P concentrations, indicating the underlying influence of soil pH-related processes on soil C-N-P stoichiometry. Indeed, soil pH values are measures of soil acidity/alkalinity which mainly reflects long-term effect of climate. The close relationship between soil C-N-P stoichiometry and soil pH values was indeed a direct reflection of the climatic effect.

In the less arid sites such as semi-arid and dry sub-humid ecosystems, there are more C and N available for plants and microorganisms to uptake. Simultaneously, available P kept at a relatively high level can couple C, N and P to biological processes. In comparison, soil organic C, total N and P all decline with aridity, but in different degrees, resulting in low C:P and N:P ratios, but high C:N ratio at arid sites. Likewise, N:P ratio declines when inorganic N and P are considered instead of total N and P. Similar patterns were found for soil C-N-P stoichiometry in relation to temperature. The observed stoichiometric changes suggest that, in response to increasing aridity and warming, plant growth is limited by the decoupling of soil biogeochemistry: C and N become uncoupled from P which is no longer reliant on C and N levels. Soil N also becomes uncoupled from C in arid and warm conditions, implying an imbalance of soil C-N-P concentrations that could constrain plant and microbial activity and thus biomass production and decomposition. Our observations here accord with the results of short-term drought-induced decoupling C-N-P under rainfall manipulation^48^,^49^.

Although soil organic C, total N and P are strongly associated with each other in our studied sites (Supplementary materials, Fig. S16), the observed aridity- and temperature-related changes in stoichiometric C-N-P ratios indicate that future climatic changes, particularly increasing drought and global warming, will unbalance soil C-N-P concentrations and therefore likely uncouple their biogeochemical cycles at least in drylands of northern China. The low growth rate of plants and low primary production in arid ecosystems reflects a fragile coupling between these biogeochemical cycles, especially under rapid climate change in these regions. In contrast, dry sub-humid regions (aridity index >0.65) might be more resistant to drought and these ecosystems will probably more easily recover to previous ecosystem stoichiometry post-drought. It should be noted that C, N and P cycling in arid systems can still be coupled (despite being fragile), even when their ratios change, as long as cycling of these elements is controlled by biological processes. Changes in C:N:P stoichiometry do not necessarily imply less control by biological processes. The diminishing interaction between plants and soil life with increasing drought and temperature in drylands of northern China, however, could upset soil C-N-P balance and reduce the resilience of arid ecosystems in a changing world, pushing currently arid regions into full desert aridity.

## Conclusion

Our 3500-km transect study in 65 dryland sites of northern China’s steppe, to our knowledge, was the first to explore how aridity and warming influence soil C-N-P balance in meadow steppe, typical steppe and desert steppe, all of which are expected to experience drier and warmer climates^1^,^2^. Our results reveal that the predicted increasing drought and warming in drylands can lower soil C and N concentrations that are primarily linked to biological processes. However, soil P availability is not affected by aridity although soil total P declines with aridity, suggesting that increasing mechanical rock weathering might have exceeded decreasing biological weathering (decomposition of organic matter) with aridity. The different trends of soil total and available P with temperature strengthened this idea. The changes in soil C, N and P across the rainfall and temperature gradient could be attributed to aridity/temperature variations and the rainfall/temperature-induced change in soils and vegetation (e.g. soil pH and vegetation cover), whether through direct or indirect effects. Reflecting by stoichiometric ratio changes, soil C-N-P concentrations are unbalanced and plant production declines accordingly. Therfore, C-N-P cycles might be interrupted in more arid and warm ecosystems, likely negatively affecting biogeochemical-controlled ecosystem functions despite plants have the adaptive capacity. The similar responses of soil C-N-P to declined rainfall and increased temperature suggest that the disturbance of soil nutrient balance in dryland ecosystems could worsen due to the global warming-induced increase in drought.

## Methods

### Experiment design

Sixty-five grassland sites located in Inner Mongolia, China, were selected for this study. The sites were chosen along a 3500-km-long transect, located in arid, semi-arid and dry sub-humid areas covering a wide spectrum which differs in climates, soil types, vegetation cover and species richness. Mean annual temperature was 2.1 °C, with a tendency for higher temperatures in the south-west. Mean annual precipitation decreased from 457 mm yr^−1^ to 154 mm yr^−1^, reflecting the transition from a humid to an arid climate. The corresponding summer rainfall (May to September) in plant growth season decreased from 404 to 133 mm yr^−1^. Due to the close relation among aridity, precipitation, and temperature, this aridity gradient can also be considered as a precipitation gradient or a temperature gradient.

### Soil sample collection and measurement

The spatial geographical coordinates (longitude and latitude) and altitude of each site were obtained by GPS. We established a 30 m × 30 m plot representative of the dominant vegetation at each site and collected 15 soil samples at the 0–30 cm layer in the plot of each site in 2013 with a soil core (5 cm in diameter). Soil samples were taken to the laboratory, air-dried and sieved for laboratory analyses of C, N, P and pH. Soil organic C was analyzed by colorimetry following dichromate oxidation when boiling with a mixture of potassium dichromate and sulphuric acid. Total N was measured using the Kjeldahl acid-digestion method. Total P was determined by the sulfuric acid hydrolysis procedure. Available soil N, determined as the sum of ammonium, nitrate and dissolved organic nitrogen, was analyzed colorimetrically using a continuous-flow ion auto-analyzer (Scalar SAN^plus^ segmented flow analyzer, the Netherlands). Olsen inorganic P was measured by extracting soil with 0.5 M NaHCO_3_ at pH 8.5. The HCl-P fraction was determined as described by Tiessen and Moir^50^. The available P referred to inorganic P which was calculated as the sum of Olsen inorganic P and HCl-P. Soil pH was determined in 1:2.5 (w/v) soil water suspensions using a glass electrode. Soil types, classified according to the FAO-UNESCO classification system, were derived from the Harmonised World Soil Database version 1.21 released in 2012 by FAO.

### Climatic data

We used aridity for our modelling because it is a fundamental driver of biological and geochemical processes in drylands. The aridity index (AI) is the ratio of mean annual precipitation (MAP) to mean annual potential evapotranspiration (MAE). MAP values were obtained from the WorldClim Global Climate Data (http://WorldClim.org), for years 1950–2000, by using R package ‘*raster*’. Potential evapo-transpiration (PET) layers were estimated on a monthly average basis by the Global-PET (http://www.cgiar-csi.org) and were aggregated to MAE. The AI decreases as aridity increases and AI is strongly related to climatic characters such as temperature and rainfall (Supplementary materials, Fig. S11). Our studied sites cover an aridity gradient from AI = 0.167 to AI = 0.604. We derived other climatic variables such as mean annual temperature from WorldClim Global Climate Data. Because soil nutrients bare more associated with current year climate than with mean climate, we derived the mean temperature and summed rainfall from May to August in 2013, i.e., the climatic variables in growth season prior to field sampling (ftp://ftp.ncdc.noaa.gov/pub/data/gsod/).

### Statistical analysis

By using mean and standard errors, all soil variables at each site were averaged to obtain site-level estimates for statistical analysis. Probability of fit to normal distribution was tested by Shapiro–Wilk test. One-way analysis of variance was used to test the effect of soil types on soil C, N and P characteristics. When the difference was significant, post-hoc multiple comparisons were subsequently made using the Tukey–Kramer test. The relationships of independent variables (aridity index, longitude, latitude, altitude, growth-season temperature and precipitation in 2013) with the dependent variables (soil organic C, total N, total P, available N, available P, and their stoichiometric ratios of C:N, C:P and N:P) were explored by using either linear or curvilinear (quadratic) regressions. N:P ratios, calculated from total N and total P, or from available N and available P, were both considered in the analysis. To achieve normality, stoichiometric ratios of C, N and P were log-transformed before analyses. Multiple regression analysis with backward stepwise procedure was used to determine the best model (based on the Akaike information criterion [AIC]) and examine the overall patterns of response of soil C, N and P and their ratios to climatic (temperature and precipitation), soil pH and plant cover. Regression models were developed with increasing numbers of independent variables. The models labelled ‘T + R + ST + pH + PC’ include temperature, precipitation, soil type, soil pH and plant cover as explanatory variables (Table 2). For each of soil C, N and P and their ratios, two models were compared, including: 1) factors selected by the stepwise procedure, and 2) all factors. The values of the regression coefficients of the best model were used to compare the contribution of each variable to the soil variables (‘strength of effects’). The results were interpreted by examining the relative influence (or contribution) of each predictor from the best model based on MASS package. Statistical analyses were conducted with R 3.2.2.

## Additional Information

**How to cite this article**: Jiao, F. *et al.* Increasing aridity, temperature and soil pH induce soil C-N-P imbalance in grasslands. *Sci. Rep.* **6**, 19601; doi: 10.1038/srep19601 (2016).

## Supplementary Material

Supplementary Information

## Figures and Tables

**Table 1:** The soil characteristics of 65 grassland sampling sites.

Soil type	AI	AB	pH	SOC	TN	TP	AN	AP	TC:N	TC:P	TN:P	AN:P
Anthrosol	0.20	194 ± 22b	8.0 ± 0.1a	0.1 ± 0.02c	0.01 ± 0.00d	0.2 ± 0.01b	5.2 ± 0.3ab	3.8 ± 0.3ab	10.0 ± 2.0a	7 ± 2c	0.7 ± 0.04c	1.4 ± 0.1abc
Arenosol	0.31	201 ± 39b	7.6 ± 0.1ab	1.3 ± 0.3abc	0.11 ± 0.02abcd	0.3 ± 0.03b	7.6 ± 0.6ab	4.0 ± 0.4ab	21.2 ± 2.8a	35 ± 5bc	2.9 ± 0.5bc	3.1 ± 0.5abc
Calcisol	0.25	169 ± 36c	7.9 ± 0.1a	0.7 ± 0.1c	0.06 ± 0.01cd	0.3 ± 0.02b	6.1 ± 0.3ab	4.1 ± 0.3ab	21.1 ± 6.7a	27 ± 4bc	2.2 ± 0.3bc	1.9 ± 0.3abc
Cambisol	0.42	149 ± 35c	6.4 ± 0.1c	2.5 ± 0.3ab	0.26 ± 0.02ab	0.4 ± 0.02b	12.4 ± 2.0a	1.6 ± 0.2b	9.7 ± 0.6a	66 ± 6ab	6.9 ± 0.4ab	7.9 ± 0.9a
Chernozem	0.48	269 ± 11bc	6.5 ± 0.1bc	2.5 ± 0.3ab	0.23 ± 0.03abc	0.3 ± 0.01b	11.6 ± 1.1ab	2.8 ± 0.9ab	10.7 ± 0.1a	95 ± 11a	8.9 ± 1.1a	5.6 ± 2.2ab
Gleysol	0.17	20 ± 3d	8.5 ± 0.1a	0.4 ± 0.03c	0.02 ± 0.00d	0.2 ± 0.04b	6.0 ± 0.4ab	3.4 ± 0.6ab	17.4 ± 0.8a	18 ± 3c	1.0 ± 0.2c	1.9 ± 0.2abc
Greyzem	0.42	325 ± 19bc	7.6 ± 0.02ab	1.4 ± 0.1abc	0.11 ± 0.01abcd	0.7 ± 0.13a	2.5 ± 0.1b	2.8 ± 0.3ab	12.9 ± 0.3a	21 ± 7bc	1.6 ± 0.5c	0.9 ± 0.1bc
Kastanozem	0.33	253 ± 27c	7.5 ± 0.1abc	1.2 ± 0.1abc	0.11 ± 0.01abcd	0.3 ± 0.01b	8.7 ± 0.6ab	3.5 ± 0.2ab	12.3 ± 0.3a	44 ± 2bc	3.8 ± 0.2bc	4.4 ± 0.6abc
Leptosol	0.37	280 ± 21bc	7.7 ± 0.1a	1.2 ± 0.03abc	0.10 ± 0.00bcd	0.4 ± 0.01b	3.0 ± 0.4b	5.1 ± 0.2ab	12.3 ± 0.5a	33 ± 1bc	2.7 ± 0.1bc	0.6 ± 0.1c
Phaeozem	0.48	296 ± 61bc	6.6 ± 0.1bc	2.9 ± 0.2a	0.28 ± 0.02a	0.5 ± 0.05ab	9.2 ± 0.6ab	4.3 ± 0.4ab	10.2 ± 0.3a	67 ± 4ab	6.7 ± 0.5ab	3.1 ± 0.5abc
Solonchack	0.44	462 ± 33a	6.6 ± 0.02bc	2.0 ± 0.1abc	0.18 ± 0.01abcd	0.5 ± 0.01ab	12.9 ± 0.7a	5.8 ± 1.2a	11.2 ± 0.3a	45 ± 1bc	4.0 ± 0.1bc	2.4 ± 0.4abc
mean	0.35	241 ± 19	7.4 ± 0.1	1.5 ± 0.1	0.14 ± 0.01	0.4 ± 0.01	8.2 ± 0.3	3.8 ± 0.1	14.5 ± 1.0a	44 ± 2.0	4.0 ± 0.2	3.5 ± 0.3

Data are means ± 1 SE. The soils are classified according to the Harmonised World Soil Database (HWSD, version 1.21) released by FAO. Different letters indicate significant (*P* < 0.05) differences among soil types. Abbreviations: AI, aridity index (unitless); AB, aboveground biomass (g m^−2^); SOC, soil organic carbon (%); TN, soil total nitrogen (%); TP, soil total phosphorus (%); AN, available nitrogen (ppm); AP, available phosphorus (ppm); TC:N, ratio of total carbon to nitrogen; TC:P, ratio of total carbon to phosphorus; TN:P, ratio of total nitrogen to phosphorus; AN:P, ratio of available nitrogen to phosphorus.

**Table 2:** *r*^2^ values in multiple regression analyses of soil carbon, nitrogen, phosphorus and their ratios.

Model	SOC	TN	TP	AN	AP	TC:N	TC:P	TN:P	AN:P
Aridity (A)	0.567***	0.520***	0.331***	0.184***	0.001 ns	0.194***	0.324***	0.326***	0.100**
Temperature (T)	0.761***	0.742***	0.306***	0.161***	0.040 ns	0.353***	0.555***	0.568***	0.133**
Rainfall (R)	0.419***	0.370***	0.275***	0.136**	0.008 ns	0.116**	0.224***	0.212***	0.063*
Soil type (ST)	0.336***	0.282**	0.092 ns	0.073 ns	0.034 ns	0.124 ns	0.270**	0.218**	0.003 ns
Soil pH (pH)	0.620***	0.642***	0.154***	0.308***	0.098*	0.372***	0.542***	0.567***	0.268***
Plant cover (PC)	0.088*	0.079*	0.073*	0.005 ns	0.016 ns	0.020 ns	0.030 ns	0.033 ns	0.009 ns
T + R	0.765***	0.747***	0.336***	0.161**	0.041 ns	0.359***	0.558***	0.576***	0.119**
T + R + ST	0.779***	0.785***	0.419***	0.244***	0.010 ns	0.371***	0.592***	0.572***	0.125*
T + R + ST + pH	0.802***	0.827***	0.426***	0.321***	0.112 ns	0.460***	0.663***	0.665***	0.268**
Overall	0.810***	0.831**	0.427***	0.289**	0.097 ns	0.458***	0.670***	0.667***	0.239*
stepAIC	−213	−181	−225	−205	−189	−260	−221	−177	−136
TotalAIC	−211	−170	−214	−200	−180	−252	−210	−170	−126

ns (not significant, *P* > 0.05), *(*P* < 0.05), **(*P* < 0.01), ***(*P* < 0.001). The ‘+’ includes the relevant predictors and their interactions. The models labelled ‘Overall’ includes temperature, rainfall, soil pH and plant cover as explanatory variables. StepAIC: AIC value of model selected by the stepwise procedure. TotalAIC: AIC value of model with all parameters. AIC is the Akaike Information Criterion.
