# Supplementary material for: Increasing aridity, temperature and soil pH induce soil C-N-P imbalance in grasslands
Source: Sci Rep. 2016 Jan 21;6:19601. doi: 10.1038/srep19601 (PMC4726211; doi:10.1038/srep19601)
Supplement: Supplementary Information [file srep19601-s1.pdf]

Supplementary Information for

Increasing aridity, temperature and soil pH induce soil C-N-P imbalance in grasslands

Feng Jiao[1,2], Xin-Rong Shi[1,2], Feng-Peng Han[1,2], Zhi-You Yuan[1,2,*]

Corresponding to ZYY: zyyuan@ms.iswc.ac.cn

The PDF includes:

Additional captions for Figures 1 to 16

Supplementary Figures 1 to 16

Supplementary R codes

Additional captions for Figures 1 to 12

Supplementary Fig. S1. Soil C, N, P and pH in relation to aridity index. Aridity index is the ratio of precipitation to potential evapotranspiration. The solid dark grey lines represent the fitted quadratic regressions. The values of soil C, N, P and pH are log-transformed. R^2, proportion of variance explained. Generalized climate classification scheme for aridity values on the top is based on UNEP.

Supplementary Fig. S2. Soil C, N, P and pH in relation to mean temperature from May to August, 2013. The solid dark grey lines represent the fitted quadratic regressions. The values of soil C, N, P and pH are log-transformed. R^2, proportion of variance explained.

Supplementary Fig. S3. Soil C, N, P and pH in relation to total rainfall from May to August, 2013. The solid dark grey lines represent the fitted quadratic regressions. The values of soil C, N, P and pH are log-transformed. R^2, proportion of variance explained.

Supplementary Fig. S4. Soil C, N, and P in relation to soil pH values. The solid dark grey lines represent the fitted quadratic regressions. The values of soil C, N, and P are log-transformed. R^2, proportion of variance explained.

Supplementary Fig. S5. Soil organic carbon in relation to latitude, longitude, altitude and soil moisture. The solid dark grey lines represent the fitted regressions. R^2, proportion of variance explained. The values of soil organic carbon are log-transformed.

Supplementary Fig. S6. Soil total nitrogen in relation to latitude, longitude, altitude and soil moisture. The solid dark grey lines represent the fitted regressions. R^2, proportion of variance explained. The values of soil total nitrogen are log-transformed.

Supplementary Fig. S7. Soil total phosphorus in relation to latitude, longitude, altitude and soil moisture. The solid dark grey lines represent the fitted regressions. R^2, proportion of variance explained. The values of soil total phosphorus are log-transformed.

Supplementary Fig. S8. Soil available nitrogen in relation to latitude, longitude, altitude and soil moisture. The solid dark grey lines represent the fitted regressions. R^2, proportion of variance explained. The values of soil available nitrogen are log-transformed.

Supplementary Fig. S9. Soil available phosphorus in relation to latitude, longitude, altitude and soil moisture. The solid dark grey lines represent the fitted regressions. R^2, proportion of variance explained. The values of soil available phosphorus are log-transformed.

Supplementary Fig. S10. Soil pH values in relation to latitude, longitude, altitude and soil moisture. The solid dark grey lines represent the fitted regressions. R^2, proportion of variance explained.

Supplementary Fig. S11. Soil carbon to total nitrogen ratios in relation to latitude, longitude, altitude and soil moisture. The solid dark grey lines represent the fitted regressions. R^2, proportion of variance explained. The ratios of soil carbon to total nitrogen are log-transformed.

Supplementary Fig. S12. Soil total carbon to phosphorus ratios in relation to latitude, longitude, altitude and soil moisture. The solid dark grey lines represent the fitted regressions. R^2, proportion of variance explained. The ratios of soil total carbon to phosphorus are log-transformed.

Supplementary Fig. S13. Soil total nitrogen to phosphorus ratios in relation to latitude, longitude, altitude and soil moisture. The solid dark grey lines represent the fitted regressions. R^2, proportion of variance explained. The ratios of soil total nitrogen to phosphorus are log-transformed.

Supplementary Fig. S14. Soil available nitrogen to phosphorus ratios in relation to latitude, longitude, altitude and soil moisture. The solid dark grey lines represent the fitted regressions. R^2, proportion of variance explained. The ratios of soil available nitrogen to phosphorus are log-transformed.

Supplementary Fig. S15. Relations among independent variables of climates and soil characters. The solid dark grey lines represent the fitted regressions. R^2, proportion of variance explained.

Supplementary Fig. S16. Relations among dependent variables of soil characters. The solid dark grey lines represent the fitted regressions. R^2, proportion of variance explained.

Supplementary Figures 1 to 16

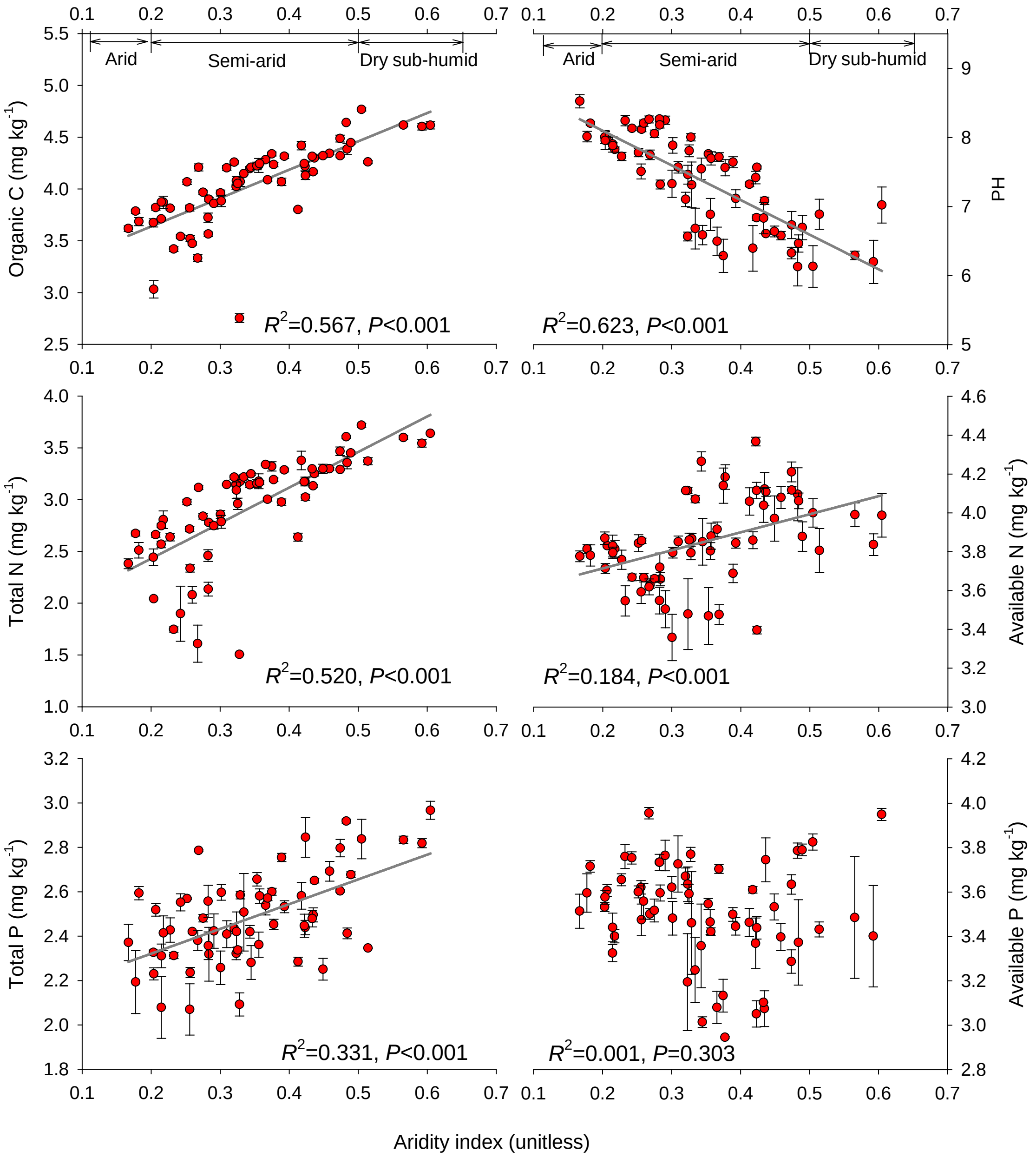

Supplementary Fig. S1. Soil C, N, P and pH in relation to aridity index. Aridity index is the ratio of precipitation to potential evapotranspiration. The solid dark grey lines represent the fitted quadratic regressions. The values of soil C, N, P and pH are log-transformed. R^2, proportion of variance explained. Generalized climate classification scheme for aridity values on the top is based on UNEP.

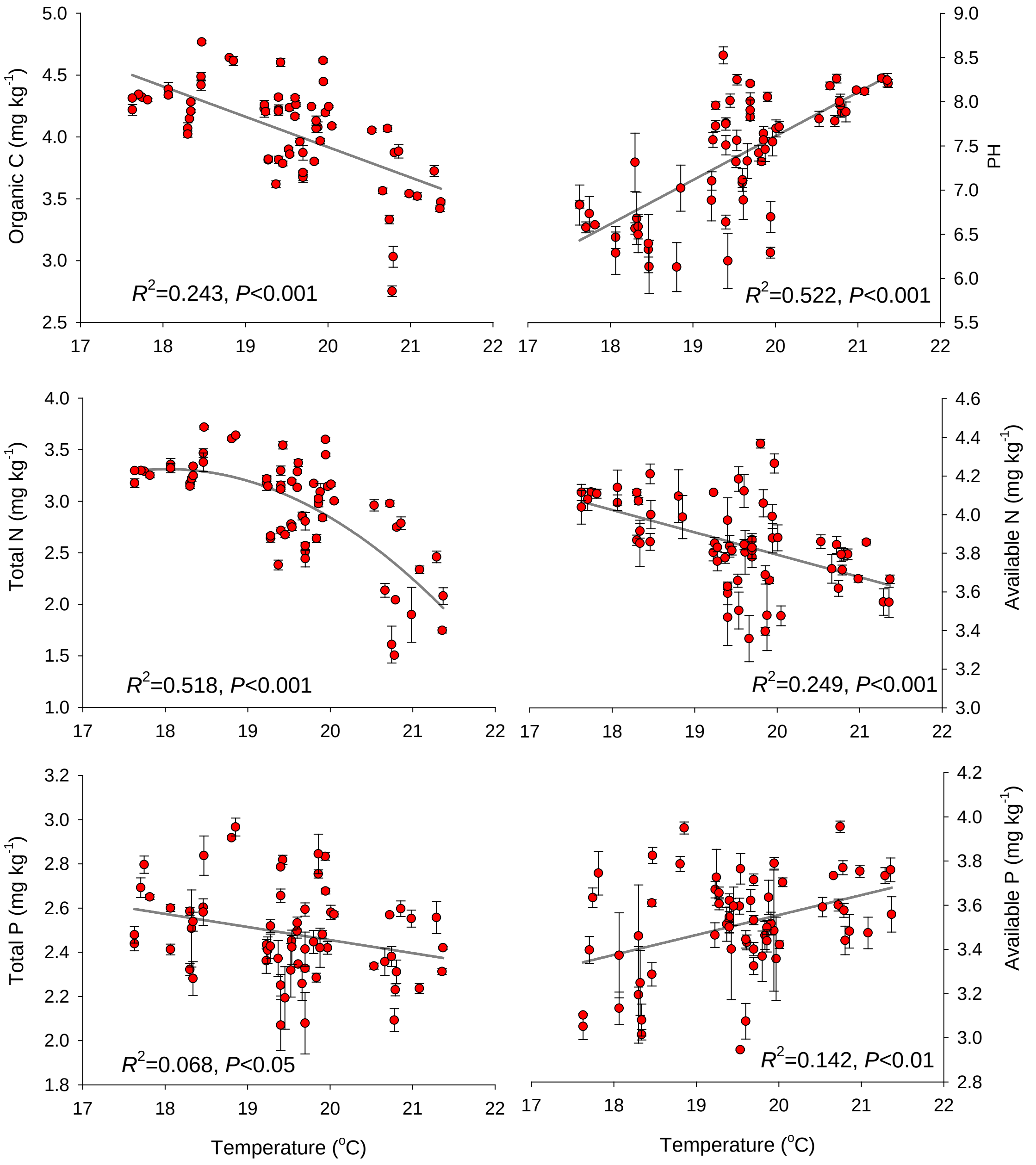

Supplementary Fig. S2. Soil C, N, P and pH in relation to mean temperature from May to August, 2013. The solid dark grey lines represent the fitted quadratic regressions. The values of soil C, N, P and pH are log-transformed. R^2, proportion of variance explained.

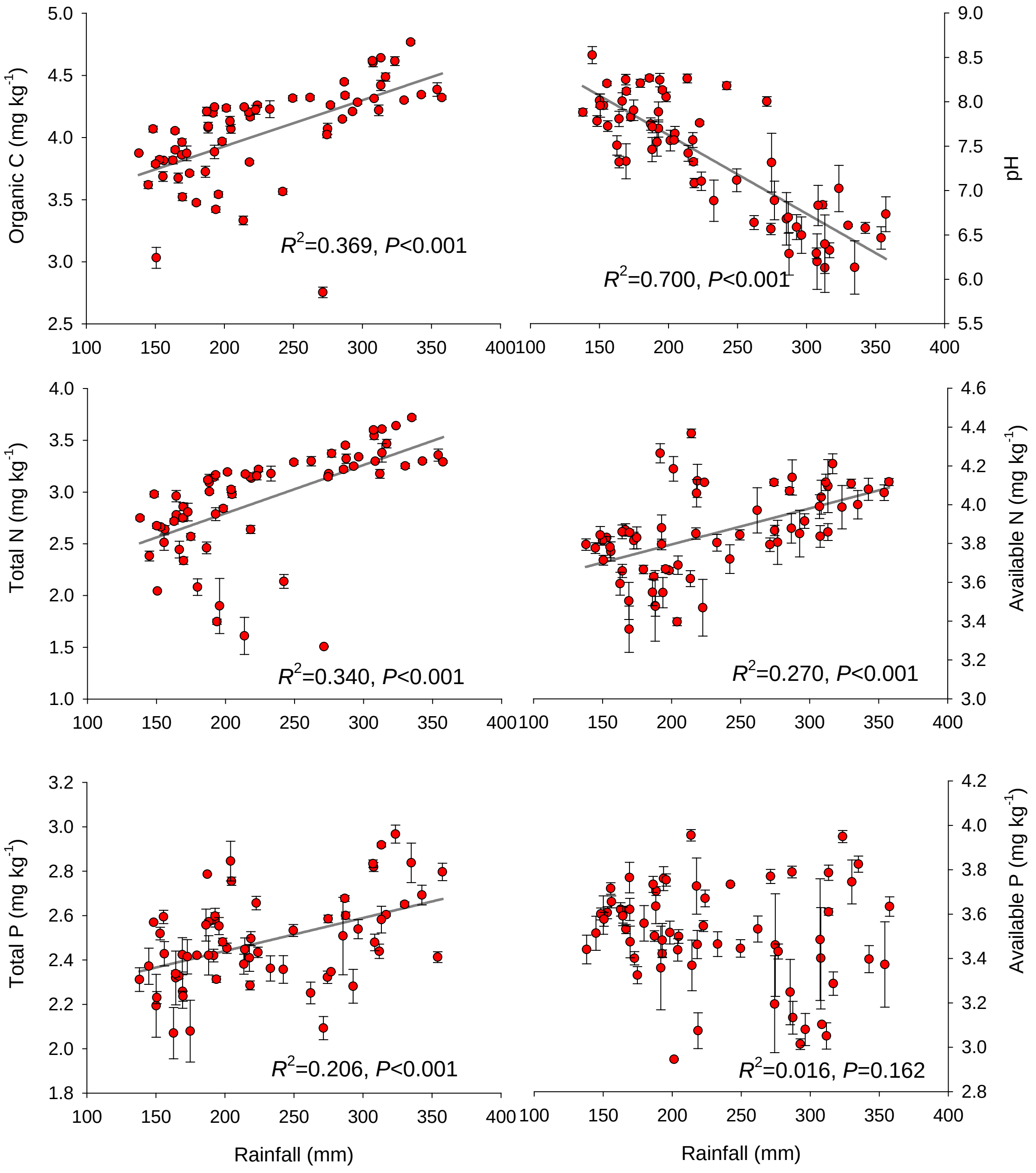

Supplementary Fig. S3. Soil C, N, P and pH in relation to total rainfall from May to August, 2013. The solid dark grey lines represent the fitted quadratic regressions. The values of soil C, N, P and pH are log-transformed. R^2, proportion of variance explained.

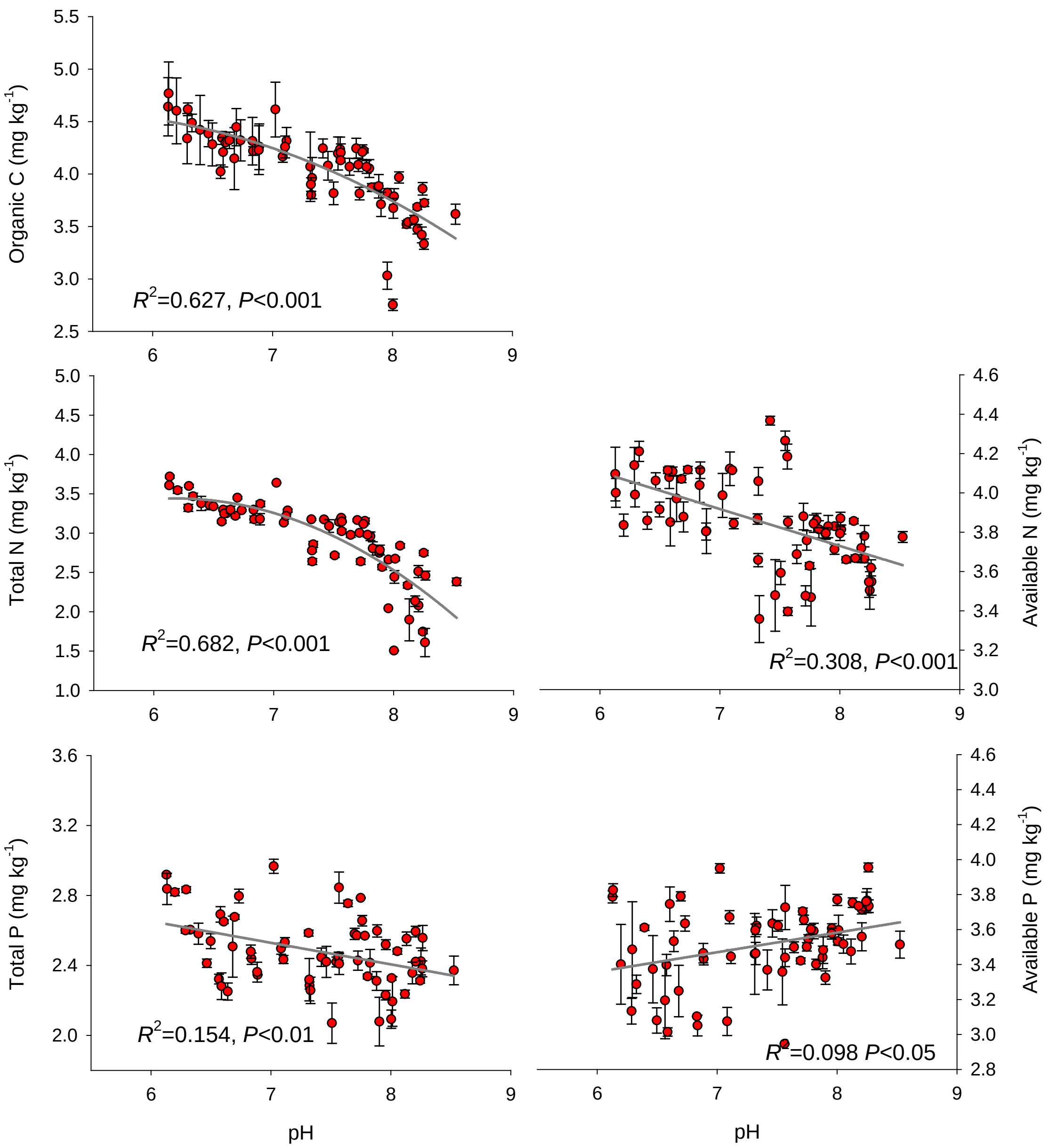

Supplementary Fig. S4. Soil C, N and P in relation to soil pH values. The solid dark grey lines represent the fitted quadratic regressions. The values of soil C, N and P are log-transformed. R^2, proportion of variance explained.

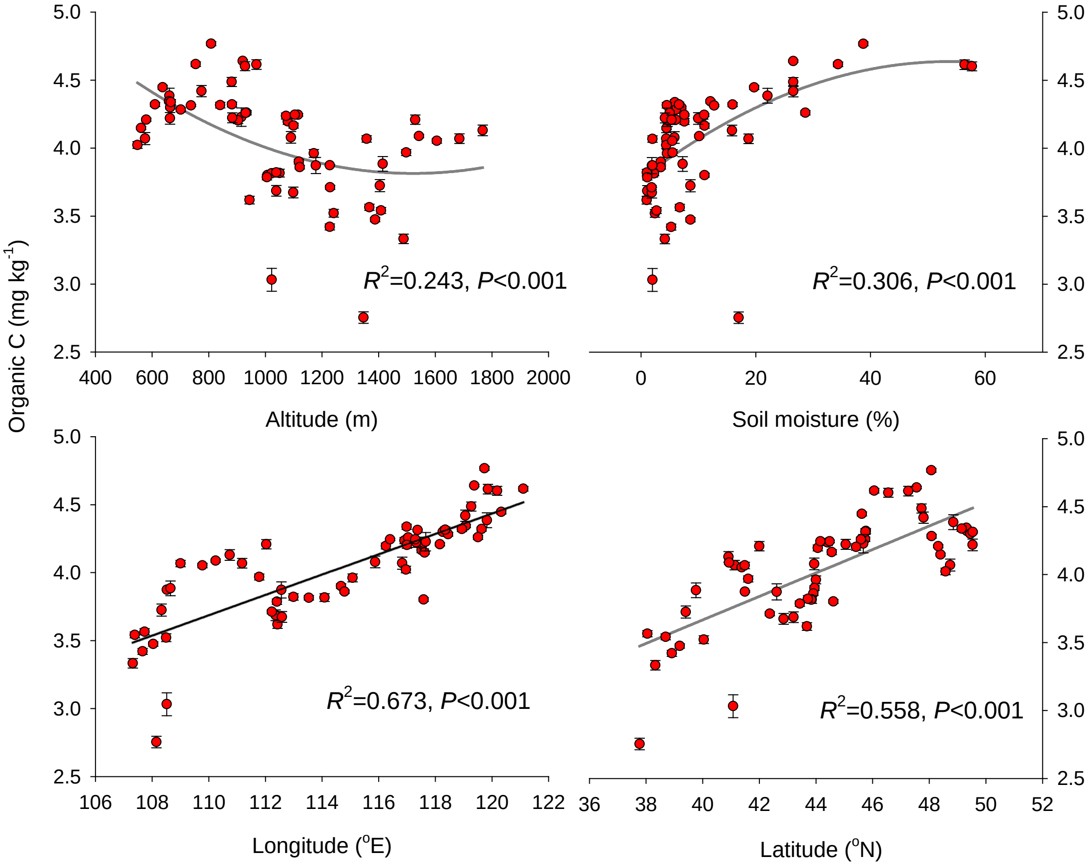

Supplementary Fig. S5. Soil organic carbon in relation to latitude, longitude, altitude and soil moisture. The solid dark grey lines represent the fitted regressions. R^2, proportion of variance explained. The values of soil organic carbon are log-transformed.

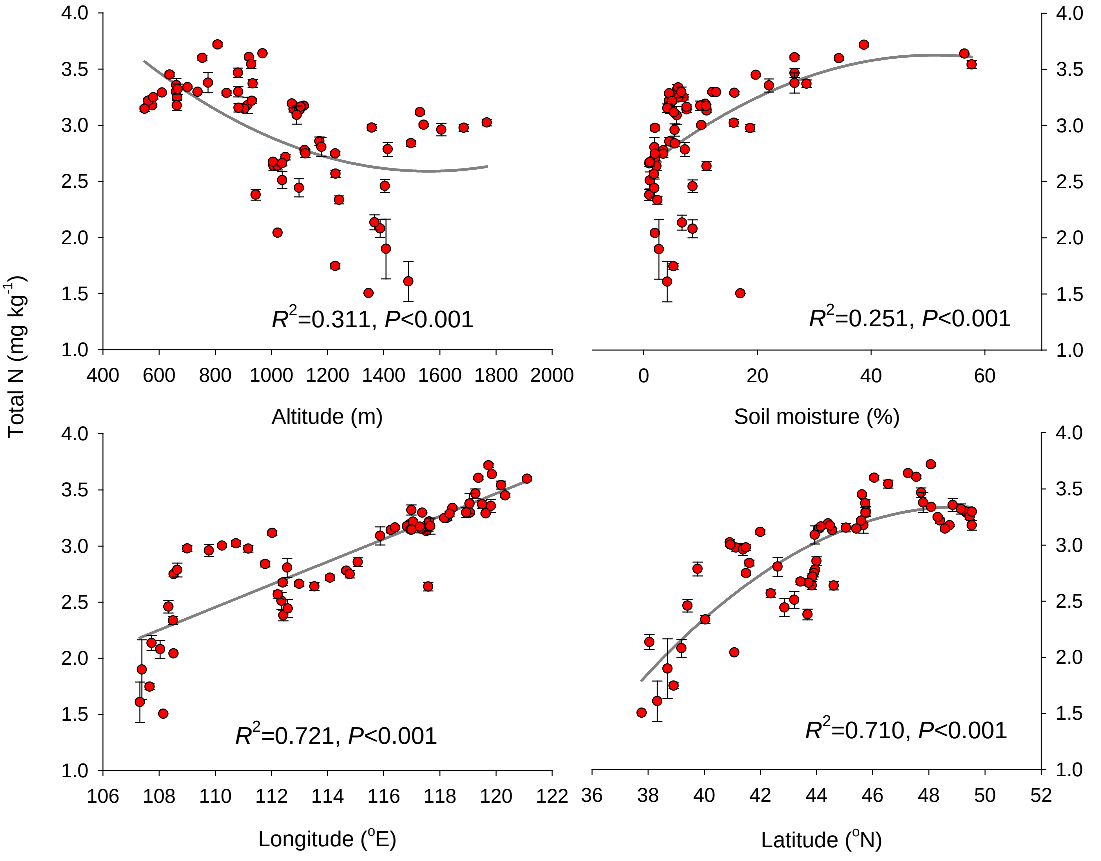

Supplementary Fig. S6. Soil total nitrogen in relation to latitude, longitude, altitude and soil moisture. The solid dark grey lines represent the fitted regressions. R^2, proportion of variance explained. The values of soil total nitrogen are log-transformed.

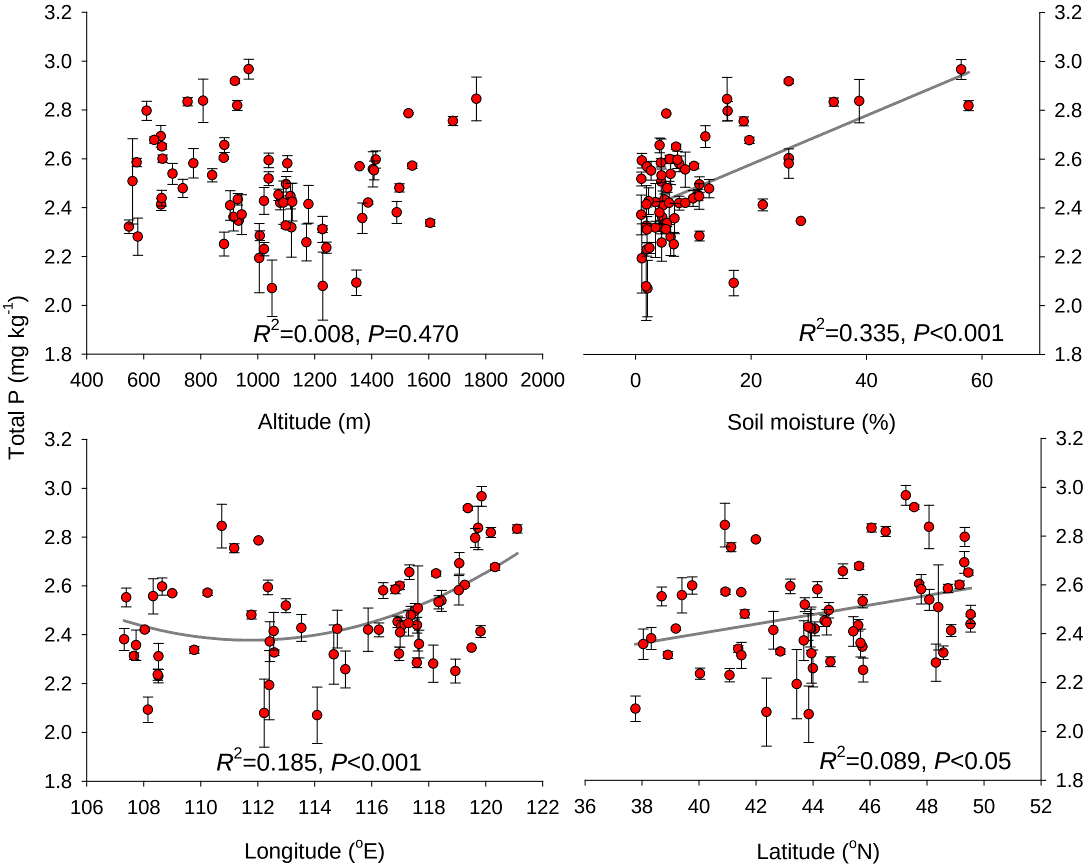

Supplementary Fig. S7. Soil total phosphorus in relation to latitude, longitude, altitude and soil moisture. The solid dark grey lines represent the fitted regressions. R^2, proportion of variance explained. The values of soil total phosphorus are log-transformed.

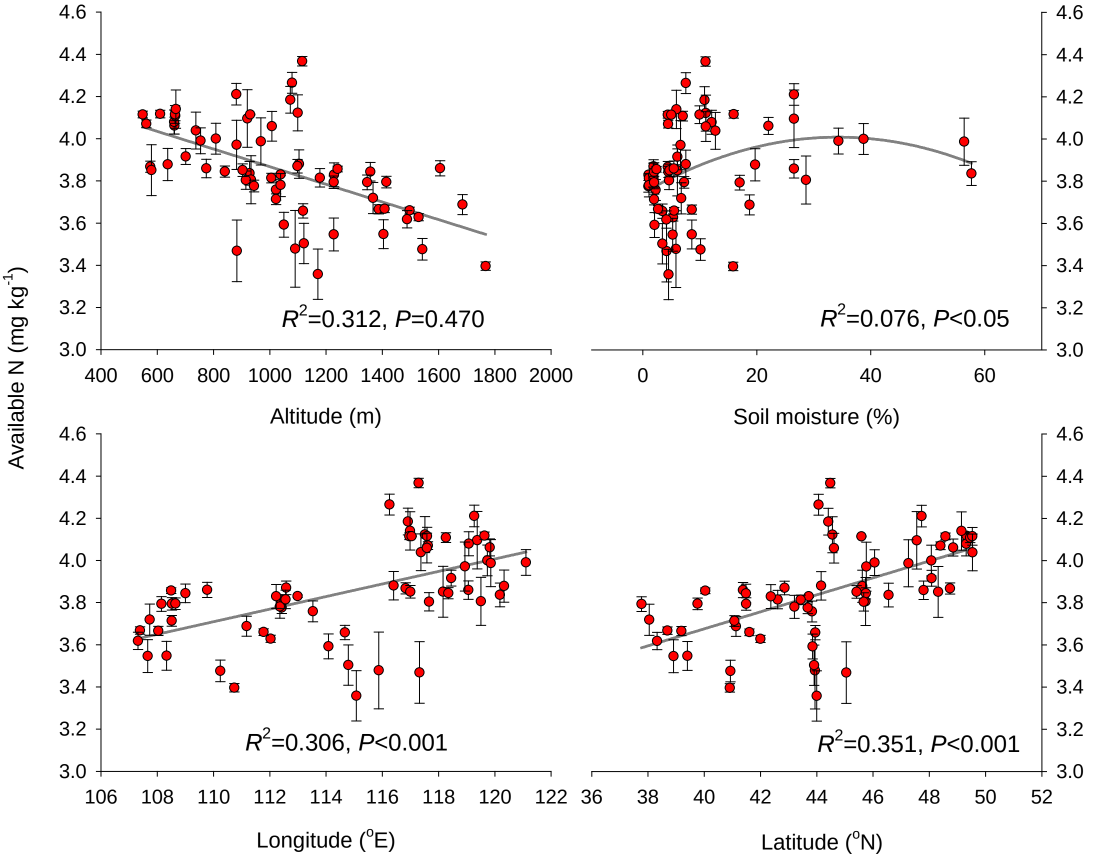

Supplementary Fig. S8. Soil available nitrogen in relation to latitude, longitude, altitude and soil moisture. The solid dark grey lines represent the fitted regressions. R^2, proportion of variance explained. The values of soil available nitrogen are log-transformed.

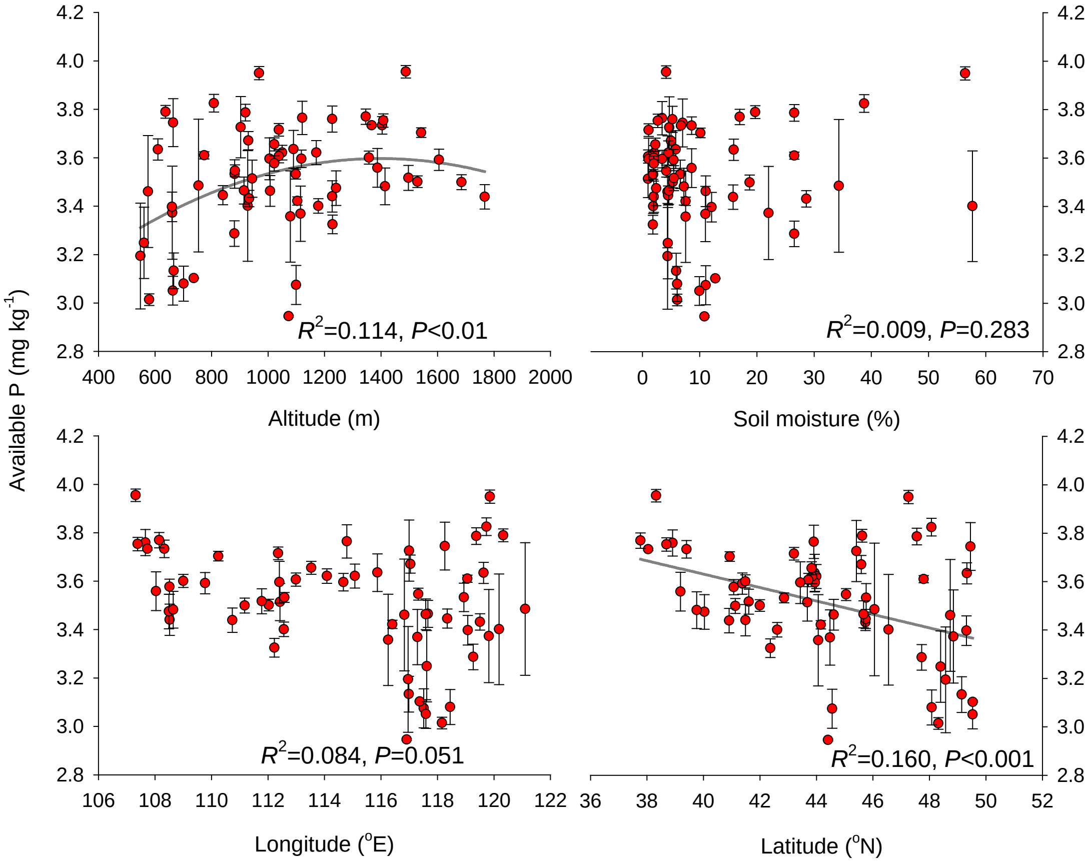

Supplementary Fig. S9. Soil available phosphorus in relation to latitude, longitude, altitude and soil moisture. The solid dark grey lines represent the fitted regressions. R^2, proportion of variance explained. The values of soil available phosphorus are log-transformed.

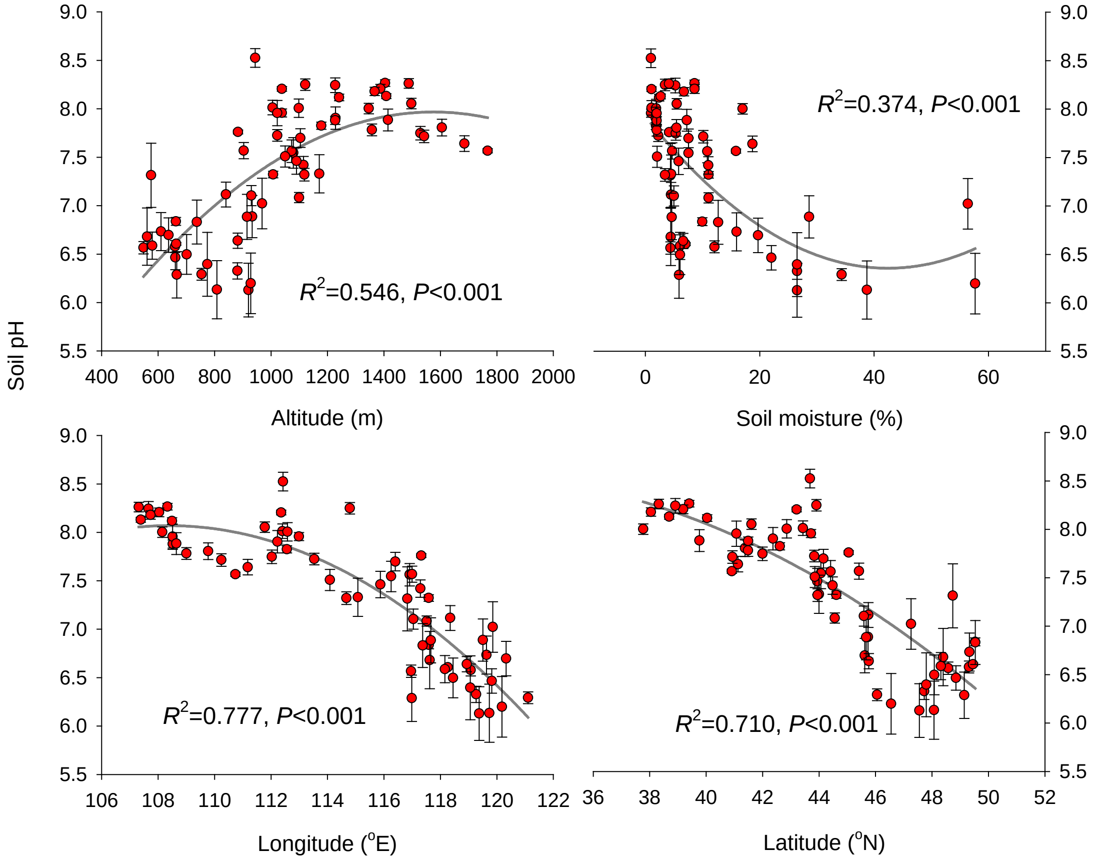

Supplementary Fig. S10. Soil pH values in relation to latitude, longitude, altitude and soil moisture. The solid dark grey lines represent the fitted regressions. R^2, proportion of variance explained.

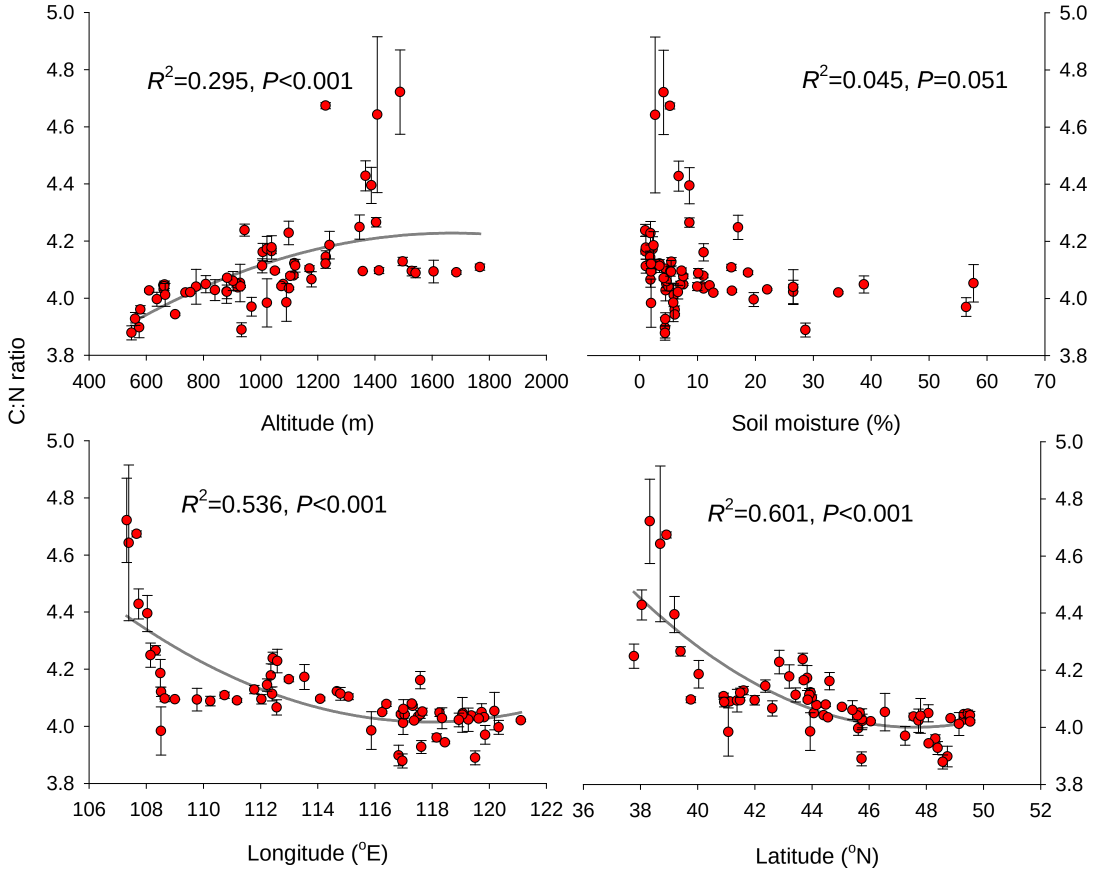

Supplementary Fig. S11. Soil carbon to total nitrogen ratios in relation to latitude, longitude, altitude and soil moisture. The solid dark grey lines represent the fitted regressions. R^2, proportion of variance explained. The ratios of soil carbon to total nitrogen are log-transformed.

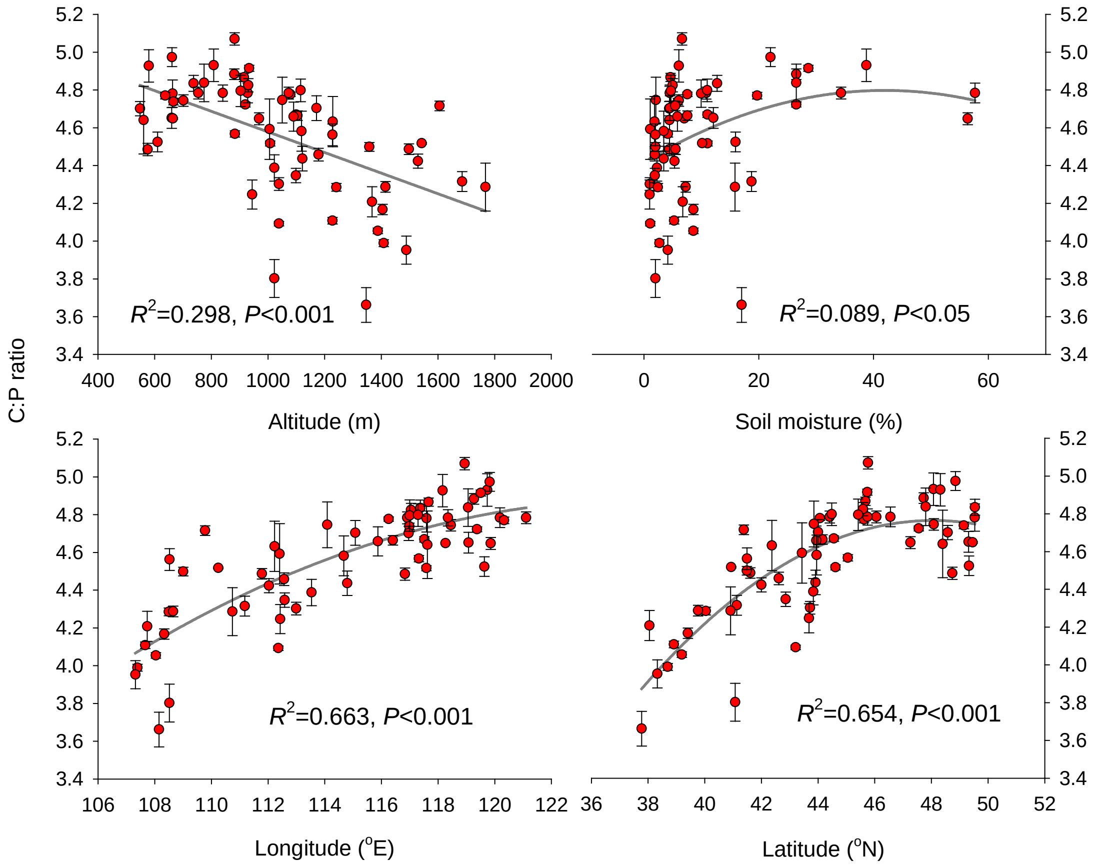

Supplementary Fig. S12. Soil total carbon to phosphorus ratios in relation to latitude, longitude, altitude and soil moisture. The solid dark grey lines represent the fitted regressions. R^2, proportion of variance explained. The ratios of soil total carbon to phosphorus are log-transformed.

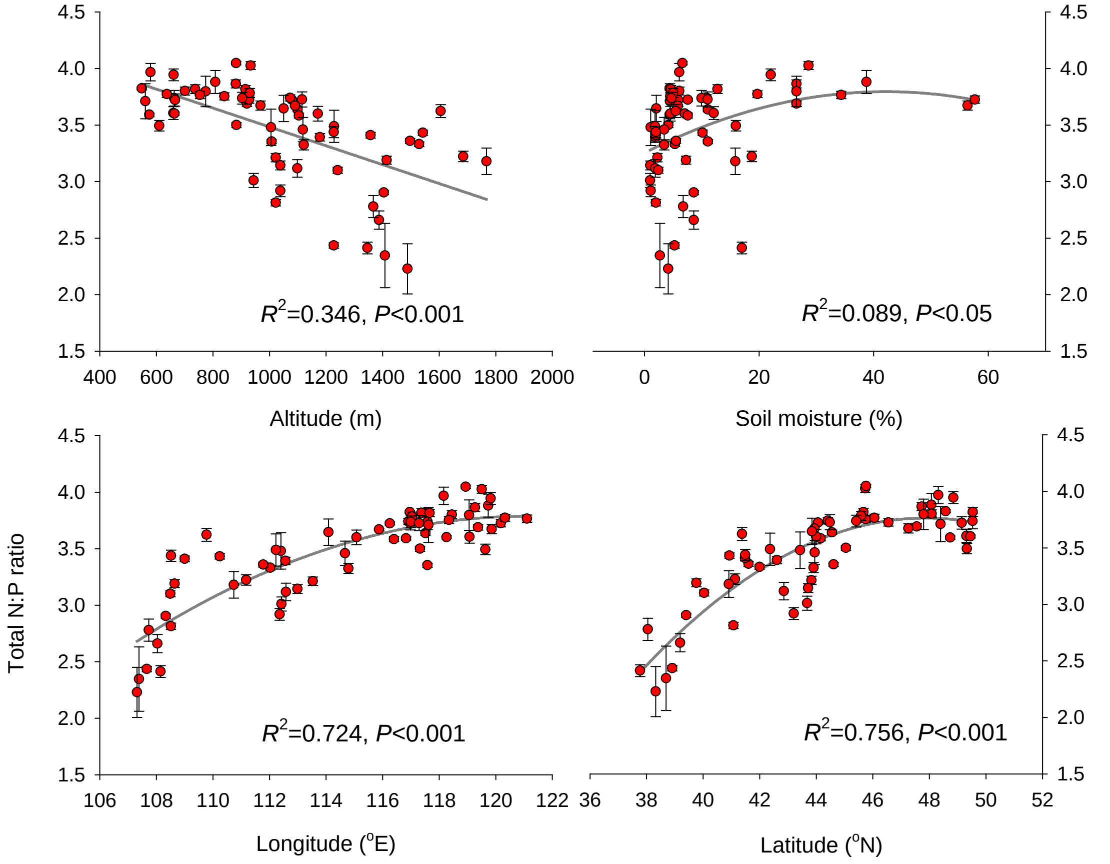

Supplementary Fig. S13. Soil total nitrogen to phosphorus ratios in relation to latitude, longitude, altitude and soil moisture. The solid dark grey lines represent the fitted regressions. R^2, proportion of variance explained. The ratios of soil total nitrogen to phosphorus are log-transformed.

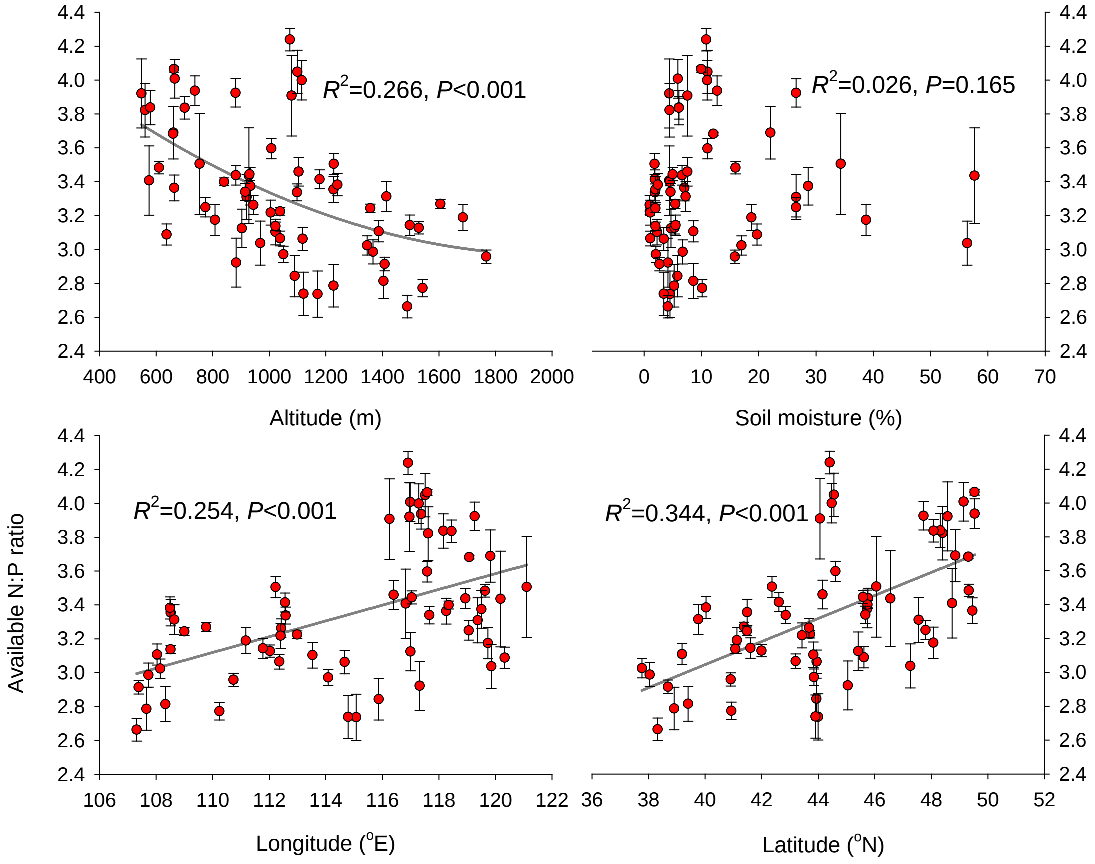

Supplementary Fig. S14. Soil available nitrogen to phosphorus ratios in relation to latitude, longitude, altitude and soil moisture. The solid dark grey lines represent the fitted regressions. R^2, proportion of variance explained. The ratios of soil available nitrogen to phosphorus are log-transformed.

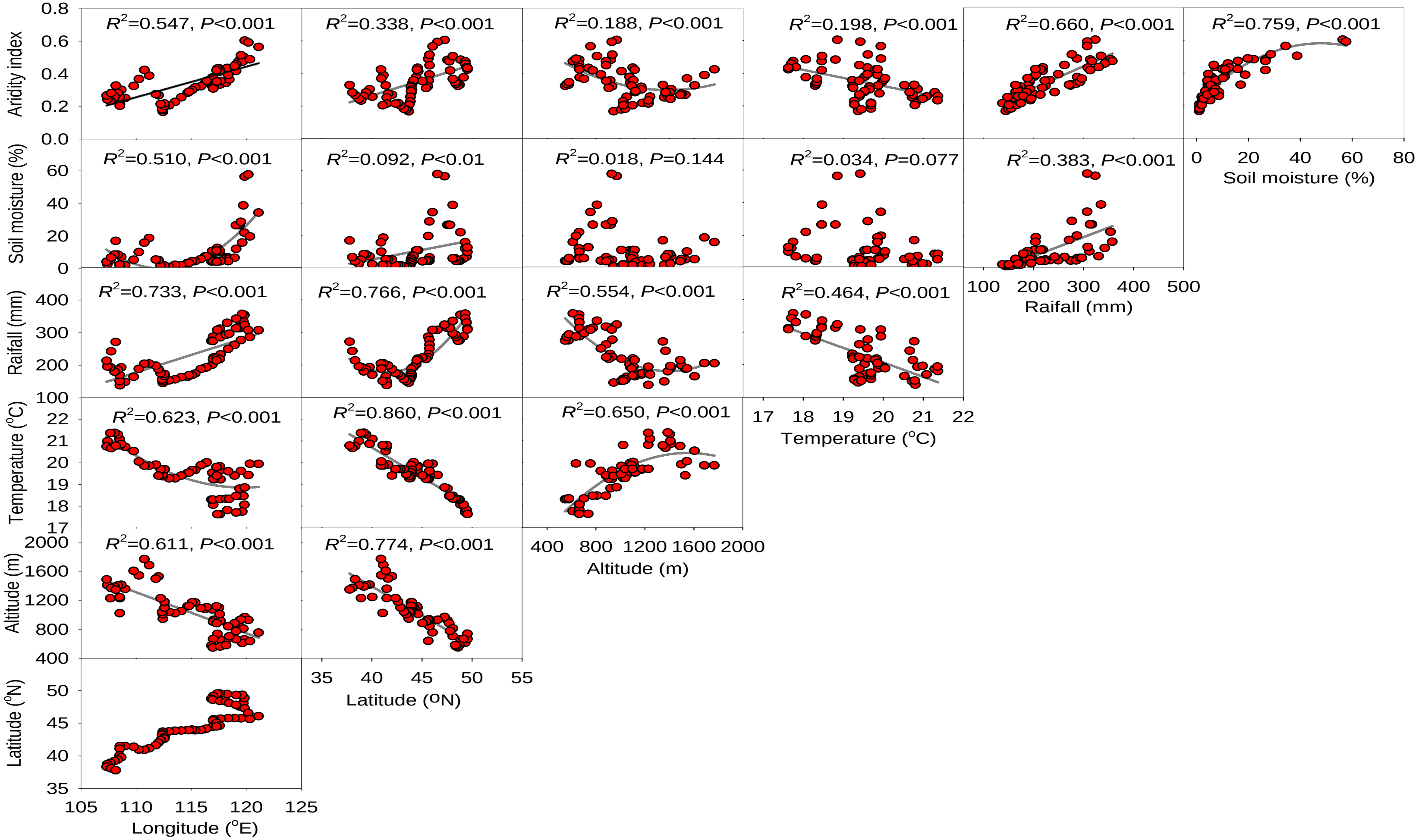

Supplementary Fig. S15. Relations among independent variables of climates and soil characters. The solid dark grey lines represent the fitted regressions. R^2, proportion of variance explained.

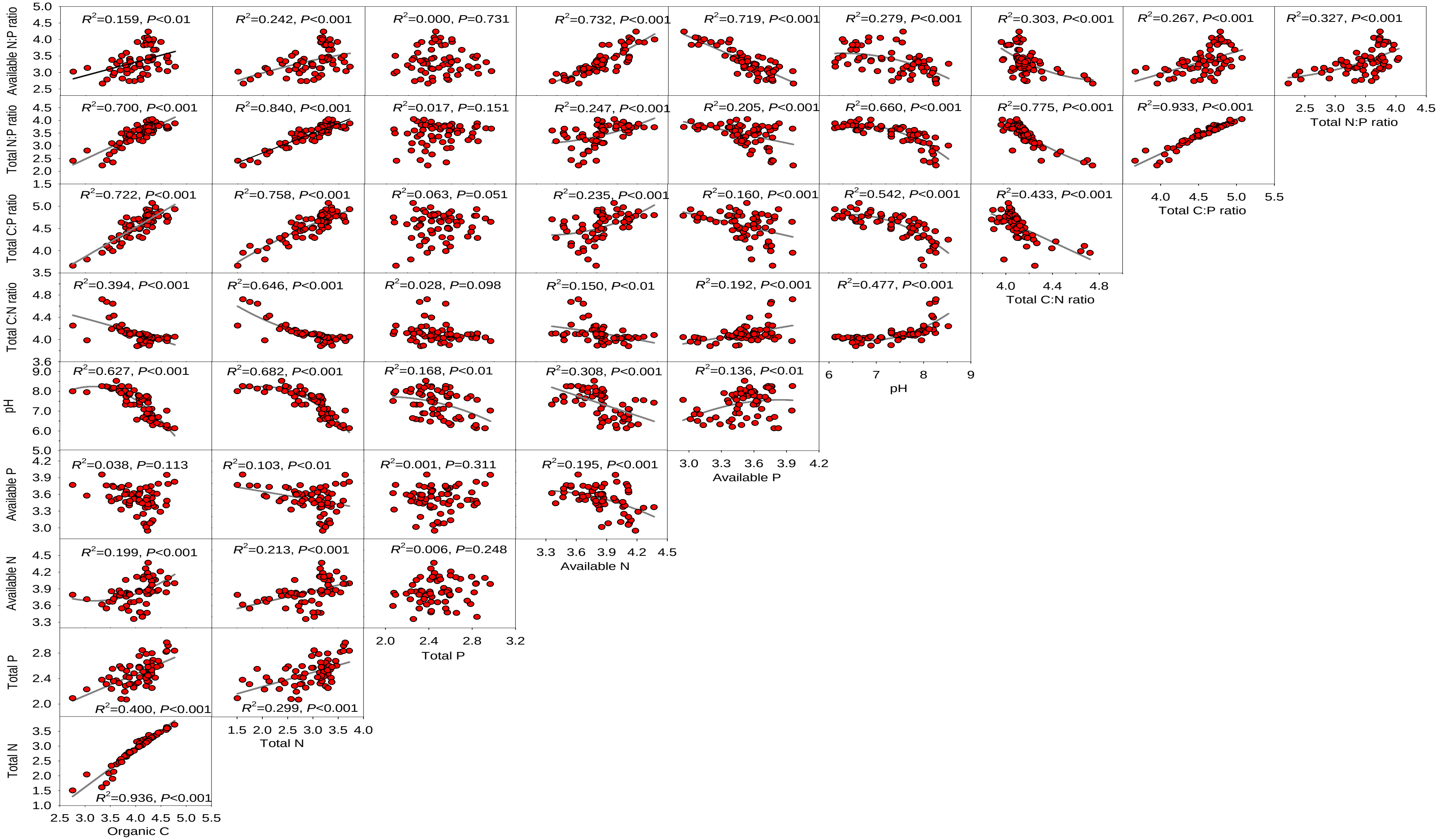

Supplementary Fig. S16. Relations among dependent variables of soil characters. The solid dark grey lines represent the fitted regressions. R^2, proportion of variance explained.

Supplementary R codes

```
###### install packages
install.packages(c("rgeos", "ncdf","ncdf4", "igraph", "snow", "tcltk", "rasterVis", "raster", "rgdal", "MASS"))
library(raster)
library(rgdal)

####### decriptive information
### read in coordinate data
location<-read.csv(file.choose(),header=T,sep=",",row.names=1)


##2.5 resolution
worldprec2.5<-getData('worldclim', var = "prec", res = 2.5)# import precipitation data
worldbio2.5<-getData('worldclim', var = "bio", res = 2.5)# import Bioclimatic variables
worldtmin2.5<-getData('worldclim', var = "tmin", res = 2.5)#import min temperaure data
worldtmax2.5<-getData('worldclim', var = "tmax", res = 2.5)#import max temperaure data
worldtmean2.5<-getData('worldclim', var = "tmean", res = 2.5)#import mean temperaure data
worldalt2.5<-getData('worldclim', var = "alt", res = 2.5)#import altitude data

# locating sampling sites information
prec2.5<-extract(worldprec2.5, location)
bio2.5<-extract(worldbio2.5, location)
tmin2.5<-extract(worldtmin2.5, location)
tmax2.5<-extract(worldtmax2.5, location)
tmean2.5<-extract(worldtmean2.5, location)
alt2.5<-extract(worldalt2.5, location)


write.csv(prec2.5,"prec2.5.csv",row.names=F)
write.csv(bio2.5,"bio2.5.csv",row.names=F)
write.csv(tmin2.5,"tmin2.5.csv",row.names=F)
write.csv(tmax2.5,"tmax2.5.csv",row.names=F)
write.csv(tmean2.5,"tmean2.5.csv",row.names=F)
write.csv(alt2.5,"alt2.5.csv",row.names=F)


## 0.5 resolutiion
site1<-getData('worldclim', var='prec', res=0.5, lon=116.25277, lat=44.06392)
```

```
site2<-getData('worldclim', var='prec', res=0.5, lon=116.90558, lat=44.41)
site3<-getData('worldclim', var='prec', res=0.5, lon=117.51397, lat=44.55632)
site4<-getData('worldclim', var='prec', res=0.5, lon=119.373306, lat=47.551117)
site5<-getData('worldclim', var='prec', res=0.5, lon=119.264191, lat=47.723309)
site6<-getData('worldclim', var='prec', res=0.5, lon=119.73151, lat=48.07299)
site7<-getData('worldclim', var='prec', res=0.5, lon=119.817734, lat=48.846348)
site8<-getData('worldclim', var='prec', res=0.5, lon=119.632126, lat=49.326477)
site9<-getData('worldclim', var='prec', res=0.5, lon=119.072586, lat=49.312557)
site10<-getData('worldclim', var='prec', res=0.5, lon=118.260658, lat=49.451397)
site11<-getData('worldclim', var='prec', res=0.5, lon=117.589828, lat=49.530769)
site12<-getData('worldclim', var='prec', res=0.5, lon=117.367755, lat=49.53241)
site13<-getData('worldclim', var='prec', res=0.5, lon=116.983505, lat=49.142448)
site14<-getData('worldclim', var='prec', res=0.5, lon=116.828949, lat=48.738518)
site15<-getData('worldclim', var='prec', res=0.5, lon=116.959953, lat=48.576267)
site16<-getData('worldclim', var='prec', res=0.5, lon=117.620857, lat=48.394867)
site17<-getData('worldclim', var='prec', res=0.5, lon=118.15609, lat=48.314449)
site18<-getData('worldclim', var='prec', res=0.5, lon=118.446877, lat=48.07901)
site19<-getData('worldclim', var='prec', res=0.5, lon=119.056717, lat=47.79668)
site20<-getData('worldclim', var='prec', res=0.5, lon=119.85186, lat=47.25846)
site21<-getData('worldclim', var='prec', res=0.5, lon=120.181435, lat=46.552937)
site22<-getData('worldclim', var='prec', res=0.5, lon=121.105415, lat=46.05547)
site23<-getData('worldclim', var='prec', res=0.5, lon=120.32798, lat=45.61841)
site24<-getData('worldclim', var='prec', res=0.5, lon=119.501938, lat=45.735249)
site25<-getData('worldclim', var='prec', res=0.5, lon=118.934464, lat=45.758511)
site26<-getData('worldclim', var='prec', res=0.5, lon=118.34692, lat=45.74453)
site27<-getData('worldclim', var='prec', res=0.5, lon=117.661896, lat=45.668709)
site28<-getData('worldclim', var='prec', res=0.5, lon=117.04174, lat=45.58729)
site29<-getData('worldclim', var='prec', res=0.5, lon=116.992615, lat=45.41214)
site30<-getData('worldclim', var='prec', res=0.5, lon=117.320274, lat=45.049538)
site31<-getData('worldclim', var='prec', res=0.5, lon=117.582794, lat=44.609718)
site32<-getData('worldclim', var='prec', res=0.5, lon=117.28849, lat=44.480217)
site33<-getData('worldclim', var='prec', res=0.5, lon=116.399864, lat=44.155289)
site34<-getData('worldclim', var='prec', res=0.5, lon=115.87172, lat=43.93767)
site35<-getData('worldclim', var='prec', res=0.5, lon=115.342239, lat=43.898647)
site36<-getData('worldclim', var='prec', res=0.5, lon=115.072075, lat=43.999531)
site37<-getData('worldclim', var='prec', res=0.5, lon=114.669479, lat=43.946178)
site38<-getData('worldclim', var='prec', res=0.5, lon=114.792302, lat=43.907854)
site39<-getData('worldclim', var='prec', res=0.5, lon=114.08559, lat=43.85021)
site40<-getData('worldclim', var='prec', res=0.5, lon=113.530098, lat=43.828537)
site41<-getData('worldclim', var='prec', res=0.5, lon=112.98703, lat=43.71168)
site42<-getData('worldclim', var='prec', res=0.5, lon=112.421425, lat=43.676239)
site43<-getData('worldclim', var='prec', res=0.5, lon=112.40471, lat=43.427586)
site44<-getData('worldclim', var='prec', res=0.5, lon=112.35817, lat=43.20352)
site45<-getData('worldclim', var='prec', res=0.5, lon=112.58075, lat=42.852158)
```

```
site46<-getData('worldclim', var='prec', res=0.5, lon=112.562225, lat=42.608479)
site47<-getData('worldclim', var='prec', res=0.5, lon=112.222656, lat=42.368118)
site48<-getData('worldclim', var='prec', res=0.5, lon=112.028015, lat=41.996109)
site49<-getData('worldclim', var='prec', res=0.5, lon=111.777435, lat=41.607098)
site50<-getData('worldclim', var='prec', res=0.5, lon=111.17422, lat=41.12784)
site51<-getData('worldclim', var='prec', res=0.5, lon=110.7355, lat=40.907)
site52<-getData('worldclim', var='prec', res=0.5, lon=110.23671, lat=40.93141)
site53<-getData('worldclim', var='prec', res=0.5, lon=109.77082, lat=41.37786)
site54<-getData('worldclim', var='prec', res=0.5, lon=108.99704, lat=41.480907)
site55<-getData('worldclim', var='prec', res=0.5, lon=108.51762, lat=41.48585)
site56<-getData('worldclim', var='prec', res=0.5, lon=108.513287, lat=41.071325)
site57<-getData('worldclim', var='prec', res=0.5, lon=108.492218, lat=40.035061)
site58<-getData('worldclim', var='prec', res=0.5, lon=108.64634, lat=39.76187)
site59<-getData('worldclim', var='prec', res=0.5, lon=108.32901, lat=39.40164)
site60<-getData('worldclim', var='prec', res=0.5, lon=108.03331, lat=39.188469)
site61<-getData('worldclim', var='prec', res=0.5, lon=107.660149, lat=38.90781)
site62<-getData('worldclim', var='prec', res=0.5, lon=107.384178, lat=38.691818)
site63<-getData('worldclim', var='prec', res=0.5, lon=107.31415, lat=38.32182)
site64<-getData('worldclim', var='prec', res=0.5, lon=107.727966, lat=38.04464)
site65<-getData('worldclim', var='prec', res=0.5, lon=108.147415, lat=37.76812)


install.packages("gsod", repos="http://R-Forge.R-project.org")
install.packages("weatherData", repos="http://R-Forge.R-project.org")
library(weatherData)
install.packages("cropData", repos="http://R-Forge.R-project.org")
library(cropData)
install.packages(c("maps","vegan","reshape"))
library(maps)
library(vegan)
library(reshape)


data(stations)
locsExtent<-c(105,125,35,50)
stationsSelected<-stationsExtent(locsExtent,stations)
plot(stationsSelected[c("LON","LAT")],pch=3,cex=.5)
map("world",add=TRUE,interior=F)


library(vegan)
library(spaa)
library(ggplot2)
```

```
####### calculate Shannon indices etc
### read in data
mydata<-read.csv(file.choose())
data65<-mydata[,c(1,5:6)]
data65<- data2mat(data65)
Shannon.Wiener <- diversity(data65, index = "shannon")
Simpson <- diversity(data65, index = "simpson")
S <- specnumber(data65)
J <- Shannon.Wiener/log(S)     ##Pielou 均匀度指数
aa<-cbind(Shannon.Wiener,Simpson,S,J)
write.csv(aa,"shanno.csv",row.names=F)

## extract PDSI
rm(list=ls())
library("RNetCDF")
PDSI_PM<-open.nc("SheffieldNature19482008.nc")
PDSI_PM_data<-read.nc(PDSI_PM)
input<-read.csv("coordinate.csv",header=T)
input$MeanPDSI<-0
for(i in 1:nrow(input))
{   m<-which(PDSI_PM_data$latitude==input$Lat[i])
    n<-which(PDSI_PM_data$longitude==input$Long[i])
    pdsi<-PDSI_PM_data$pdsi_pm[n,m,] # mean monthly PDSI from 1948 to 2008
    input$MeanPDSI[i]<-mean(pdsi)
    rm(m,n,pdsi)
}
write.csv(input,"coordinatewithPDSI.csv",row.names=F)


### regression analysis
mydata<-read.csv(file.choose())
shapiro.test(mydata$log10SOCppm);shapiro.test(mydata$log10STNppm);shapiro.test
(mydata$log10STPppm);shapiro.test(mydata$log10SANppm);shapiro.test(mydata$lo
g10SAPppm);shapiro.test(mydata$pH)
shapiro.test(mydata$log10TCN);shapiro.test(mydata$log10TCP);shapiro.test(mydata
$log10TNP); shapiro.test(mydata$log10ANP)
summary(lm(log10SOCppm~ poly(Aridity.index, 1, raw=TRUE), data=mydata))
summary(lm(log10STNppm~ poly(Aridity.index, 1, raw=TRUE), data=mydata))
summary(lm(log10STPppm~ poly(Aridity.index, 1, raw=TRUE), data=mydata))
summary(lm(log10SANppm~ poly(Aridity.index, 1, raw=TRUE), data=mydata))
summary(lm(log10SAPppm~ poly(Aridity.index, 1, raw=TRUE), data=mydata))
summary(lm(pH~ poly(Aridity.index, 1, raw=TRUE), data=mydata))
summary(lm(log10TCN~ poly(Aridity.index, 1, raw=TRUE), data=mydata))
```

```
summary(lm(log10TCP~ poly(Aridity.index, 1, raw=TRUE), data=mydata))
summary(lm(log10TNP~ poly(Aridity.index, 1, raw=TRUE), data=mydata))
summary(lm(log10ANP~ poly(Aridity.index, 1, raw=TRUE), data=mydata))
#### appendix--regression
summary(lm(log10SOCppm~ poly(latitude, 1, raw=TRUE), data=mydata))
summary(lm(log10SOCppm~ poly(longitude, 1, raw=TRUE), data=mydata))
summary(lm(log10SOCppm~ poly(alt, 1, raw=TRUE), data=mydata))
summary(lm(log10SOCppm~ poly(temp, 1, raw=TRUE), data=mydata))
summary(lm(log10SOCppm~ poly(rain, 1, raw=TRUE), data=mydata))
summary(lm(log10SOCppm~ poly(soil.moisture, 1, raw=TRUE), data=mydata))

summary(lm(log10STNppm~ poly(latitude, 1, raw=TRUE), data=mydata))
summary(lm(log10STNppm~ poly(longitude, 1, raw=TRUE), data=mydata))
summary(lm(log10STNppm~ poly(alt, 1, raw=TRUE), data=mydata))
summary(lm(log10STNppm~ poly(temp, 1, raw=TRUE), data=mydata))
summary(lm(log10STNppm~ poly(rain, 1, raw=TRUE), data=mydata))
summary(lm(log10STNppm~ poly(soil.moisture, 1, raw=TRUE), data=mydata))

summary(lm(log10STPppm~ poly(latitude, 1, raw=TRUE), data=mydata))
summary(lm(log10STPppm~ poly(longitude, 1, raw=TRUE), data=mydata))
summary(lm(log10STPppm~ poly(alt, 1, raw=TRUE), data=mydata))
summary(lm(log10STPppm~ poly(temp, 1, raw=TRUE), data=mydata))
summary(lm(log10STPppm~ poly(rain, 1, raw=TRUE), data=mydata))
summary(lm(log10STPppm~ poly(soil.moisture, 1, raw=TRUE), data=mydata))

summary(lm(log10SANppm~ poly(latitude, 1, raw=TRUE), data=mydata))
summary(lm(log10SANppm~ poly(longitude, 1, raw=TRUE), data=mydata))
summary(lm(log10SANppm~ poly(alt, 1, raw=TRUE), data=mydata))
summary(lm(log10SANppm~ poly(temp, 1, raw=TRUE), data=mydata))
summary(lm(log10SANppm~ poly(rain, 1, raw=TRUE), data=mydata))
summary(lm(log10SANppm~ poly(soil.moisture, 1, raw=TRUE), data=mydata))

summary(lm(log10SAPppm~ poly(latitude, 1, raw=TRUE), data=mydata))
summary(lm(log10SAPppm~ poly(longitude, 1, raw=TRUE), data=mydata))
summary(lm(log10SAPppm~ poly(alt, 1, raw=TRUE), data=mydata))
summary(lm(log10SAPppm~ poly(temp, 1, raw=TRUE), data=mydata))
summary(lm(log10SAPppm~ poly(rain, 1, raw=TRUE), data=mydata))
summary(lm(log10SAPppm~ poly(soil.moisture, 1, raw=TRUE), data=mydata))

summary(lm(pH~ poly(latitude, 1, raw=TRUE), data=mydata))
summary(lm(pH~ poly(longitude, 1, raw=TRUE), data=mydata))
summary(lm(pH~ poly(alt, 1, raw=TRUE), data=mydata))
summary(lm(pH~ poly(temp, 1, raw=TRUE), data=mydata))
summary(lm(pH~ poly(rain, 1, raw=TRUE), data=mydata))
```

```
summary(lm(pH~ poly(soil.moisture, 1, raw=TRUE), data=mydata))

summary(lm(log10TCN~ poly(latitude, 1, raw=TRUE), data=mydata))
summary(lm(log10TCN~ poly(longitude, 1, raw=TRUE), data=mydata))
summary(lm(log10TCN~ poly(alt, 1, raw=TRUE), data=mydata))
summary(lm(log10TCN~ poly(temp, 1, raw=TRUE), data=mydata))
summary(lm(log10TCN~ poly(rain, 1, raw=TRUE), data=mydata))
summary(lm(log10TCN~ poly(soil.moisture, 1, raw=TRUE), data=mydata))

summary(lm(log10TCP~ poly(latitude, 1, raw=TRUE), data=mydata))
summary(lm(log10TCP~ poly(longitude, 1, raw=TRUE), data=mydata))
summary(lm(log10TCP~ poly(alt, 1, raw=TRUE), data=mydata))
summary(lm(log10TCP~ poly(temp, 1, raw=TRUE), data=mydata))
summary(lm(log10TCP~ poly(rain, 1, raw=TRUE), data=mydata))
summary(lm(log10TCP~ poly(soil.moisture, 1, raw=TRUE), data=mydata))

summary(lm(log10TNP~ poly(latitude, 1, raw=TRUE), data=mydata))
summary(lm(log10TNP~ poly(longitude, 1, raw=TRUE), data=mydata))
summary(lm(log10TNP~ poly(alt, 1, raw=TRUE), data=mydata))
summary(lm(log10TNP~ poly(temp, 1, raw=TRUE), data=mydata))
summary(lm(log10TNP~ poly(rain, 1, raw=TRUE), data=mydata))
summary(lm(log10TNP~ poly(soil.moisture, 1, raw=TRUE), data=mydata))

summary(lm(log10ANP~ poly(latitude, 1, raw=TRUE), data=mydata))
summary(lm(log10ANP~ poly(longitude, 1, raw=TRUE), data=mydata))
summary(lm(log10ANP~ poly(alt, 1, raw=TRUE), data=mydata))
summary(lm(log10ANP~ poly(temp, 1, raw=TRUE), data=mydata))
summary(lm(log10ANP~ poly(rain, 1, raw=TRUE), data=mydata))
summary(lm(log10ANP~ poly(soil.moisture, 1, raw=TRUE), data=mydata))

summary(lm(latitude~ poly(longitude, 1, raw=TRUE), data=mydata))
summary(lm(alt~ poly(longitude, 1, raw=TRUE), data=mydata))
summary(lm(temp~ poly(longitude, 1, raw=TRUE), data=mydata))
summary(lm(rain~ poly(longitude, 1, raw=TRUE), data=mydata))
summary(lm(soil.moisture~ poly(longitude, 1, raw=TRUE), data=mydata))
summary(lm(Aridity.index~ poly(longitude, 1, raw=TRUE), data=mydata))

summary(lm(alt~ poly(latitude, 1, raw=TRUE), data=mydata))
summary(lm(temp~ poly(latitude, 1, raw=TRUE), data=mydata))
summary(lm(rain~ poly(latitude, 1, raw=TRUE), data=mydata))
summary(lm(soil.moisture~ poly(latitude, 1, raw=TRUE), data=mydata))
summary(lm(Aridity.index~ poly(latitude, 1, raw=TRUE), data=mydata))

summary(lm(temp~ poly(alt, 1, raw=TRUE), data=mydata))
```

```
summary(lm(rain~ poly(alt, 1, raw=TRUE), data=mydata))
summary(lm(soil.moisture~ poly(alt, 1, raw=TRUE), data=mydata))
summary(lm(Aridity.index~ poly(alt, 1, raw=TRUE), data=mydata))

summary(lm(rain~ poly(temp, 1, raw=TRUE), data=mydata))
summary(lm(soil.moisture~ poly(temp, 1, raw=TRUE), data=mydata))
summary(lm(Aridity.index~ poly(temp, 1, raw=TRUE), data=mydata))

summary(lm(soil.moisture~ poly(rain, 1, raw=TRUE), data=mydata))
summary(lm(Aridity.index~ poly(rain, 1, raw=TRUE), data=mydata))

summary(lm(Aridity.index~ poly(soil.moisture, 1, raw=TRUE), data=mydata))

mydata<-read.csv(file.choose())
mydata<-mydata[-(35),]
summary(lm(log10SOCppm~ poly(log10STNppm, 1, raw=TRUE), data=mydata))
summary(lm(log10SOCppm~ poly(log10STPppm, 1, raw=TRUE), data=mydata))
summary(lm(log10SOCppm~ poly(log10SANppm, 1, raw=TRUE), data=mydata))
summary(lm(log10SOCppm~ poly(log10SAPppm, 1, raw=TRUE), data=mydata))
summary(lm(log10SOCppm~ poly(pH, 1, raw=TRUE), data=mydata))
summary(lm(log10SOCppm~ poly(log10TCN, 1, raw=TRUE), data=mydata))
summary(lm(log10SOCppm~ poly(log10TCP, 1, raw=TRUE), data=mydata))
summary(lm(log10SOCppm~ poly(log10TNP, 1, raw=TRUE), data=mydata))
summary(lm(log10SOCppm~ poly(log10ANP, 1, raw=TRUE), data=mydata))

summary(lm(log10STNppm~ poly(log10STPppm, 1, raw=TRUE), data=mydata))
summary(lm(log10STNppm~ poly(log10SANppm, 1, raw=TRUE), data=mydata))
summary(lm(log10STNppm~ poly(log10SAPppm, 1, raw=TRUE), data=mydata))
summary(lm(log10STNppm~ poly(pH, 1, raw=TRUE), data=mydata))
summary(lm(log10STNppm~ poly(log10TCN, 1, raw=TRUE), data=mydata))
summary(lm(log10STNppm~ poly(log10TCP, 1, raw=TRUE), data=mydata))
summary(lm(log10STNppm~ poly(log10TNP, 1, raw=TRUE), data=mydata))
summary(lm(log10STNppm~ poly(log10ANP, 1, raw=TRUE), data=mydata))

summary(lm(log10STPppm~ poly(log10SANppm, 1, raw=TRUE), data=mydata))
summary(lm(log10STPppm~ poly(log10SAPppm, 1, raw=TRUE), data=mydata))
summary(lm(log10STPppm~ poly(pH, 1, raw=TRUE), data=mydata))
summary(lm(log10STPppm~ poly(log10TCN, 1, raw=TRUE), data=mydata))
summary(lm(log10STPppm~ poly(log10TCP, 1, raw=TRUE), data=mydata))
summary(lm(log10STPppm~ poly(log10TNP, 1, raw=TRUE), data=mydata))
summary(lm(log10STPppm~ poly(log10ANP, 1, raw=TRUE), data=mydata))

summary(lm(log10SANppm~ poly(log10SAPppm, 1, raw=TRUE), data=mydata))
summary(lm(log10SANppm~ poly(pH, 1, raw=TRUE), data=mydata))
```

```
summary(lm(log10SANppm~ poly(log10TCN, 1, raw=TRUE), data=mydata))
summary(lm(log10SANppm~ poly(log10TCP, 1, raw=TRUE), data=mydata))
summary(lm(log10SANppm~ poly(log10TNP, 1, raw=TRUE), data=mydata))
summary(lm(log10SANppm~ poly(log10ANP, 1, raw=TRUE), data=mydata))

summary(lm(log10SAPppm~ poly(pH, 1, raw=TRUE), data=mydata))
summary(lm(log10SAPppm~ poly(log10TCN, 1, raw=TRUE), data=mydata))
summary(lm(log10SAPppm~ poly(log10TCP, 1, raw=TRUE), data=mydata))
summary(lm(log10SAPppm~ poly(log10TNP, 1, raw=TRUE), data=mydata))
summary(lm(log10SAPppm~ poly(log10ANP, 1, raw=TRUE), data=mydata))

summary(lm(pH~ poly(log10TCN, 1, raw=TRUE), data=mydata))
summary(lm(pH~ poly(log10TCP, 1, raw=TRUE), data=mydata))
summary(lm(pH~ poly(log10TNP, 1, raw=TRUE), data=mydata))
summary(lm(pH~ poly(log10ANP, 1, raw=TRUE), data=mydata))

summary(lm(log10TCN~ poly(log10TCP, 1, raw=TRUE), data=mydata))
summary(lm(log10TCN~ poly(log10TNP, 1, raw=TRUE), data=mydata))
summary(lm(log10TCN~ poly(log10ANP, 1, raw=TRUE), data=mydata))

summary(lm(log10TCP~ poly(log10TNP, 1, raw=TRUE), data=mydata))
summary(lm(log10TCP~ poly(log10ANP, 1, raw=TRUE), data=mydata))

summary(lm(log10TNP~ poly(log10ANP, 1, raw=TRUE), data=mydata))

### multiple regression
mydata<-read.csv(file.choose())
summary(lm(log10SOCppm ~Aridity.index, data=mydata))
summary(lm(log10SOCppm ~T0509, data=mydata))
summary(lm(log10SOCppm ~P0509, data=mydata))
summary(lm(log10SOCppm ~fao, data=mydata))
summary(lm(log10SOCppm ~pH, data=mydata))
summary(lm(log10SOCppm ~AB, data=mydata))
summary(lm(log10SOCppm ~T0509+P0509, data=mydata))
summary(lm(log10SOCppm ~T0509+P0509+fao, data=mydata))
summary(lm(log10SOCppm ~T0509+P0509+fao+pH, data=mydata))
summary(lm(log10SOCppm ~T0509+P0509+fao+pH+AB, data=mydata))

summary(lm(log10STNppm ~Aridity.index, data=mydata))
summary(lm(log10STNppm ~T0509, data=mydata))
summary(lm(log10STNppm ~P0509, data=mydata))
summary(lm(log10STNppm ~fao, data=mydata))
summary(lm(log10STNppm ~pH, data=mydata))
summary(lm(log10STNppm ~AB, data=mydata))
```

```
summary(lm(log10STNppm ~T0509+P0509, data=mydata))
summary(lm(log10STNppm ~T0509+P0509+fao, data=mydata))
summary(lm(log10STNppm ~T0509+P0509+fao+pH, data=mydata))
summary(lm(log10STNppm ~T0509+P0509+fao+pH+AB, data=mydata))

summary(lm(log10STPppm ~Aridity.index, data=mydata))
summary(lm(log10STPppm ~T0509, data=mydata))
summary(lm(log10STPppm ~P0509, data=mydata))
summary(lm(log10STPppm ~fao, data=mydata))
summary(lm(log10STPppm ~pH, data=mydata))
summary(lm(log10STPppm ~AB, data=mydata))
summary(lm(log10STPppm ~T0509+P0509, data=mydata))
summary(lm(log10STPppm ~T0509+P0509+fao, data=mydata))
summary(lm(log10STPppm ~T0509+P0509+fao+pH, data=mydata))
summary(lm(log10STPppm ~T0509+P0509+fao+pH+AB, data=mydata))

summary(lm(log10SANppm ~Aridity.index, data=mydata))
summary(lm(log10SANppm ~T0509, data=mydata))
summary(lm(log10SANppm ~P0509, data=mydata))
summary(lm(log10SANppm ~fao, data=mydata))
summary(lm(log10SANppm ~pH, data=mydata))
summary(lm(log10SANppm ~AB, data=mydata))
summary(lm(log10SANppm ~T0509+P0509, data=mydata))
summary(lm(log10SANppm ~T0509+P0509+fao, data=mydata))
summary(lm(log10SANppm ~T0509+P0509+fao+pH, data=mydata))
summary(lm(log10SANppm ~T0509+P0509+fao+pH+AB, data=mydata))

summary(lm(log10SAPppm ~Aridity.index, data=mydata))
summary(lm(log10SAPppm ~T0509, data=mydata))
summary(lm(log10SAPppm ~P0509, data=mydata))
summary(lm(log10SAPppm ~fao, data=mydata))
summary(lm(log10SAPppm ~pH, data=mydata))
summary(lm(log10SAPppm ~AB, data=mydata))
summary(lm(log10SAPppm ~T0509+P0509, data=mydata))
summary(lm(log10SAPppm ~T0509+P0509+fao, data=mydata))
summary(lm(log10SAPppm ~T0509+P0509+fao+pH, data=mydata))
summary(lm(log10SAPppm ~T0509+P0509+fao+pH+AB, data=mydata))

summary(lm(log10TCN ~Aridity.index, data=mydata))
summary(lm(log10TCN ~T0509, data=mydata))
summary(lm(log10TCN ~P0509, data=mydata))
summary(lm(log10TCN ~fao, data=mydata))
summary(lm(log10TCN ~pH, data=mydata))
summary(lm(log10TCN ~AB, data=mydata))
```

```
summary(lm(log10TCN ~T0509+P0509, data=mydata))
summary(lm(log10TCN ~T0509+P0509+fao, data=mydata))
summary(lm(log10TCN ~T0509+P0509+fao+pH, data=mydata))
summary(lm(log10TCN ~T0509+P0509+fao+pH+AB, data=mydata))

summary(lm(log10TCP ~Aridity.index, data=mydata))
summary(lm(log10TCP ~T0509, data=mydata))
summary(lm(log10TCP ~P0509, data=mydata))
summary(lm(log10TCP ~fao, data=mydata))
summary(lm(log10TCP ~pH, data=mydata))
summary(lm(log10TCP ~AB, data=mydata))
summary(lm(log10TCP ~T0509+P0509, data=mydata))
summary(lm(log10TCP ~T0509+P0509+fao, data=mydata))
summary(lm(log10TCP ~T0509+P0509+fao+pH, data=mydata))
summary(lm(log10TCP ~T0509+P0509+fao+pH+AB, data=mydata))

summary(lm(log10TNP ~Aridity.index, data=mydata))
summary(lm(log10TNP ~T0509, data=mydata))
summary(lm(log10TNP ~P0509, data=mydata))
summary(lm(log10TNP ~fao, data=mydata))
summary(lm(log10TNP ~pH, data=mydata))
summary(lm(log10TNP ~AB, data=mydata))
summary(lm(log10TNP ~T0509+P0509, data=mydata))
summary(lm(log10TNP ~T0509+P0509+fao, data=mydata))
summary(lm(log10TNP ~T0509+P0509+fao+pH, data=mydata))
summary(lm(log10TNP ~T0509+P0509+fao+pH+AB, data=mydata))

summary(lm(log10ANP ~Aridity.index, data=mydata))
summary(lm(log10ANP ~T0509, data=mydata))
summary(lm(log10ANP ~P0509, data=mydata))
summary(lm(log10ANP ~fao, data=mydata))
summary(lm(log10ANP ~pH, data=mydata))
summary(lm(log10ANP ~AB, data=mydata))
summary(lm(log10ANP ~T0509+P0509, data=mydata))
summary(lm(log10ANP ~T0509+P0509+fao, data=mydata))
summary(lm(log10ANP ~T0509+P0509+fao+pH, data=mydata))
summary(lm(log10ANP ~T0509+P0509+fao+pH+AB, data=mydata))

##Regresson with AIC
tlmsoc<-lm(log10SOCppm ~Aridity.index+T0509+P0509+fao+pH+AB,
data=mydata)
summary(tlmsoc)
summary(step(tlmsoc))
drop1(step(tlmsoc))
```

```
summary(lm(log10SOCppm ~Aridity.index+T0509+fao+pH+AB, data=mydata))
library(MASS)
stepAIC(tlmsoc, direction="backward")
library(relaimpo)
calc.relimp(tlmsoc,rela=TRUE) # relative importance

tlmstn<-lm(log10STNppm ~Aridity.index+T0509+P0509+fao+pH+AB,
data=mydata)
summary(tlmstn)
summary(step(tlmstn))
drop1(step(tlmstn))

tlmstp<-lm(log10STPppm ~Aridity.index+T0509+P0509+fao+pH+AB, data=mydata)
summary(tlmstp)
summary(step(tlmstp))
drop1(step(tlmstp))

tlmsan<-lm(log10SANppm ~Aridity.index+T0509+P0509+fao+pH+AB,
data=mydata)
summary(tlmsan)
summary(step(tlmsan))
drop1(step(tlmsan))

tlmsap<-lm(log10SAPppm ~Aridity.index+T0509+P0509+fao+pH+AB,
data=mydata)
summary(tlmsap)
summary(step(tlmsap))
drop1(step(tlmsap))

tlmtcn<-lm(log10TCN ~Aridity.index+T0509+P0509+fao+pH+AB, data=mydata)
summary(tlmtcn)
summary(step(tlmtcn))
drop1(step(tlmtcn))

tlmtcp<-lm(log10TCP ~Aridity.index+T0509+P0509+fao+pH+AB, data=mydata)
summary(tlmtcp)
summary(step(tlmtcp))
drop1(step(tlmtcp))

tlmtnp<-lm(log10TNP ~Aridity.index+T0509+P0509+fao+pH+AB, data=mydata)
summary(tlmtnp)
summary(step(tlmtnp))
drop1(step(tlmtnp))
```

```
tlmanp<-lm(log10ANP ~Aridity.index+T0509+P0509+fao+pH+AB, data=mydata)
summary(tlmanp)
summary(step(tlmanp))
drop1(step(tlmanp))

## relative importance
calc.relimp(tlmsoc,rela=TRUE)
calc.relimp(tlmstn,rela=TRUE)
calc.relimp(tlmstp,rela=TRUE)
calc.relimp(tlmsan,rela=TRUE)
calc.relimp(tlmsap,rela=TRUE)
calc.relimp(tlmtcn,rela=TRUE)
calc.relimp(tlmtcp,rela=TRUE)
calc.relimp(tlmtnp,rela=TRUE)
calc.relimp(tlmanp,rela=TRUE)
```
